# Syndecan-1 Depletion Has a Differential Impact on Hyaluronic Acid Metabolism and Tumor Cell Behavior in Luminal and Triple-Negative Breast Cancer Cells

**DOI:** 10.3390/ijms22115874

**Published:** 2021-05-30

**Authors:** Sofía Valla, Nourhan Hassan, Daiana Luján Vitale, Daniela Madanes, Fiorella Mercedes Spinelli, Felipe C. O. B. Teixeira, Burkhard Greve, Nancy Adriana Espinoza-Sánchez, Carolina Cristina, Laura Alaniz, Martin Götte

**Affiliations:** 1 Laboratorio de Fisiopatología de la Hipófisis, Centro de Investigaciones Básicas y Aplicadas (CIBA), Universidad Nacional del Noroeste de la Provincia de Buenos Aires (UNNOBA), Libertad 555, Junín (B6000), Buenos Aires 2700, Argentina; sofiaavalla@gmail.com (S.V.); carolina.cristina@nexo.unnoba.edu.ar (C.C.); 2 Centro de Investigaciones y Transferencia del Noroeste de la Provincia de Buenos Aires (CITNOBA, UNNOBA-UNSAdA-CONICET), Buenos Aires 2700, Argentina; vitaledai@gmail.com (D.L.V.); fiospinelli@gmail.com (F.M.S.); 3 Department of Gynecology and Obstetrics, Münster University Hospital, Domagkstrasse 11, 48149 Münster, Germany; n_hass07@uni-muenster.de (N.H.); felipecobt@gmail.com (F.C.O.B.T.); Nancyadriana.Espinozasanchez@ukmuenster.de (N.A.E.-S.); 4 Laboratorio de Microambiente Tumoral, Centro de Investigaciones Básicas y Aplicadas (CIBA), Universidad Nacional del Noroeste de la Provincia de Buenos Aires (UNNOBA), Libertad 555, Junín (B6000), Buenos Aires 2700, Argentina; 5 Laboratorio de Inmunología de la Reproducción, Instituto de Biología y Medicina Experimental—Consejo Nacional de Investigaciones Científicas y Técnicas (IBYME-CONICET), Vuelta de Obligado 2490, Ciudad Autónoma de Buenos Aires (C1428ADN), Buenos Aires 2700, Argentina; daniela.madanes@gmail.com; 6 Department of Radiotherapy—Radiooncology, Münster University Hospital, Robert-Koch-Str. 32, 48149 Münster, Germany; greveb@uni-muenster.de

**Keywords:** syndecan-1, hyaluronic acid, breast cancer, stem cells, apoptosis, angiogenesis

## Abstract

Glycosaminoglycans (GAGs) and proteoglycans (PGs) are major components of the glycocalyx. The secreted GAG and CD44 ligand hyaluronic acid (HA), and the cell surface PG syndecan-1 (Sdc-1) modulate the expression and activity of cytokines, chemokines, growth factors, and adhesion molecules, acting as critical regulators of tumor cell behavior. Here, we studied the effect of Sdc-1 siRNA depletion and HA treatment on hallmark processes of cancer in breast cancer cell lines of different levels of aggressiveness. We analyzed HA synthesis, and parameters relevant to tumor progression, including the stem cell phenotype, Wnt signaling constituents, cell cycle progression and apoptosis, and angiogenic markers in luminal MCF-7 and triple-negative MDA-MB-231 cells. Sdc-1 knockdown enhanced HAS-2 synthesis and HA binding in MCF-7, but not in MDA-MB-231 cells. Sdc-1-depleted MDA-MB-231 cells showed a reduced CD24-/CD44+ population. Furthermore, Sdc-1 depletion was associated with survival signals in both cell lines, affecting cell cycle progression and apoptosis evasion. These changes were linked to the altered expression of KLF4, MSI2, and miR-10b and differential changes in Erk, Akt, and PTEN signaling. We conclude that Sdc-1 knockdown differentially affects HA metabolism in luminal and triple-negative breast cancer model cell lines and impacts the stem phenotype, cell survival, and angiogenic factors.

## 1. Introduction

Proteoglycans (PGs) are glycoproteins of the cell surface and extracellular matrix (ECM) that are characterized by the presence of highly negatively charged unbranched carbohydrate chains of the glycosaminoglycan (GAG) type [1], which are capable of interacting with a multitude of ligands relevant to normal physiology and tumor progression [2,3]. Depending on the composition of their repetitive disaccharide units and the absence or presence of specific sulfation modifications, GAGs comprise the families of the non-sulfated hyaluronan (HA) [4], and the protein-linked heparin/heparan sulfate (HE/HS), chondroitin sulfate/dermatan sulfate (CS/DS) and keratan sulphate (KS) [1,2,5]. Notably, a dysregulated expression of specific PGs and GAGs has been shown to contribute mechanistically to the progression of breast cancer, the most common malignancy in women [6,7,8]. Of particular relevance in this context is syndecan-1 (Sdc-1, CD138), the most highly expressed transmembrane HSPG on the surface of epithelial cells among the four members of the Sdc family [9]. Sdc-1 can bind several growth factors, chemokines, cell adhesion molecules, and ECM components, allowing for the regulation of virtually all steps of tumor progression as defined in the Hallmarks of Cancer [10]. Although some subtype-specific differences have been reported, the majority of studies on Sdc-1 expression have assigned a negative prognostic and predictive value for high Sdc-1 expression in breast cancer, suggesting that the inhibition of Sdc-1 expression and/or function could be a therapeutic approach to impair breast tumor progression [6,8,11,12,13,14]. The relevance of altered Sdc-1 expression in breast cancer has been highlighted by several functional studies: previously, we could show that in triple-negative breast cancer cells, Sdc-1 knockdown significantly downregulated the expression of several cytokines and chemokines, and decreased the constitutive activation of STAT3 and NF-κB, thereby increasing the sensitivity of breast cancer cells to radiation therapy [15]. In another study, the frequency of the CD44+/CD24- population—marking breast cancer stem cells (CSCs)—was diminished, and the expression and activation of the pro-inflammatory IL-6/STAT3 pathway reduced after Sdc-1 siRNA knockdown [16]. Moreover, in inflammatory breast cancer cells, the downregulation of Sdc-1 decreased the protein levels of IL-6, IL-8, and growth-regulated protein GRO-alpha (CXCL1), and GRO a/b/g. Importantly, the frequency of CSCs was also diminished together with the low expression of gp130, Notch-1, -2, STAT3, and NF-κB after Sdc-1 knockdown [8]. Further functional studies have demonstrated an impact of Sdc-1 on breast cancer cell motility that could be linked to the regulation of E-cadherin, Rho GTPases, and focal adhesion kinase [17], a differential regulatory impact of membrane-bound and soluble forms of Sdc-1 on breast cancer cell invasion that affected the expression of protease inhibitors [18], and a novel immunomodulatory role of tumor Sdc-1, inducing CD4+ T-cell polarization in the breast cancer microenvironment [19]. Considering that the Sdc-1 molecule is part of a complex glycocalyx and that its function is dependent on other molecules ranging from microRNAs to heparanase [10,17], alterations in its expression could also affect the function and expression of other PGs or GAGs. For example, the HA receptor CD44 is misexpressed in Sdc-1-depleted breast cancer cells [16], and MDA-MB-231 breast cancer cells depleted of beta4GalT-7, a biosynthetic enzyme generating the HS GAG attachment site, respond with a reduced expression of Sdc-1 and an upregulation of HA biosynthesis. HA is a large molecule, ranging from 10^5^ to 10^6^ Da composed of chains with 10,000 or more repetitive disaccharide units of unsulphated N-acetyl-β-D-glucosamine-D-glucuronic acid. HA is the only GAG that is synthesized at the plasma membrane, allowing its interaction with the cell surface while it is secreted to the extracellular medium [20]. The enzymes involved in its metabolism are very specific and comprise hyaluronan synthases (HASs) and hyaluronidases (Hyals), that generate an HA molecule of a specific molecular weight or fragments, which in turn have different biological activities and roles in tumor progression [21]. HA biosynthesis deregulation has been extensively observed in cancer; moreover, high levels in tumor tissues are correlated with poor prognosis of patients with different cancers, among them breast cancer [22]. In this type of cancer, the higher expression is correlated with poor carcinoma differentiation and contribution to tumor progression and aggressiveness [23,24]. Besides, several authors have demonstrated molecular mechanisms by which HA is thought to impact on tumor behavior [21], but the functional interaction with other ECM components was not extensively studied. Previous work of our group suggested that the upregulation of HA synthesis can be a result of alteration and changes in the behavior of breast cancer cells through the interference with HS biosynthesis [25]. Although HA does not bind Sdc-1 via direct interaction [5], these findings suggest that both molecules could interact functionally since they share several intracellular signaling pathways and have pleiotropic effects in tumor biology [3]. To test this hypothesis, in this work, we analyzed for the first time the effect of knocking down of Sdc-1 (Sdc-1 KD) over HA expression and function in widely used, prototypic breast cancer cell lines with different levels of aggressiveness, MCF-7 as a representative luminal cell line, and MDA-MB-231 as a representative triple-negative/basal cell line. Besides, we also analyzed whether introducing HA to the cells could reverse or exert the additive action on the phenotype of Sdc-1 KD cells. To study this, we evaluated CSC features and other hallmarks of cancer in vitro [26,27,28]. Our data suggest that Sdc-1 knockdown has a differential effect on HA metabolism in the breast cancer cell lines MCF-7 and MDA-MB-231, and impacts on the stem phenotype, cell survival, and angiogenic factor expression and secretion.

## 2. Results

### 2.1. Syndecan-1 siRNA Knockdown Has a Differential Effect on HA Metabolism and Binding in MCF-7 and MDA-MB-231 Breast Cancer Cells

**Figure 1:**
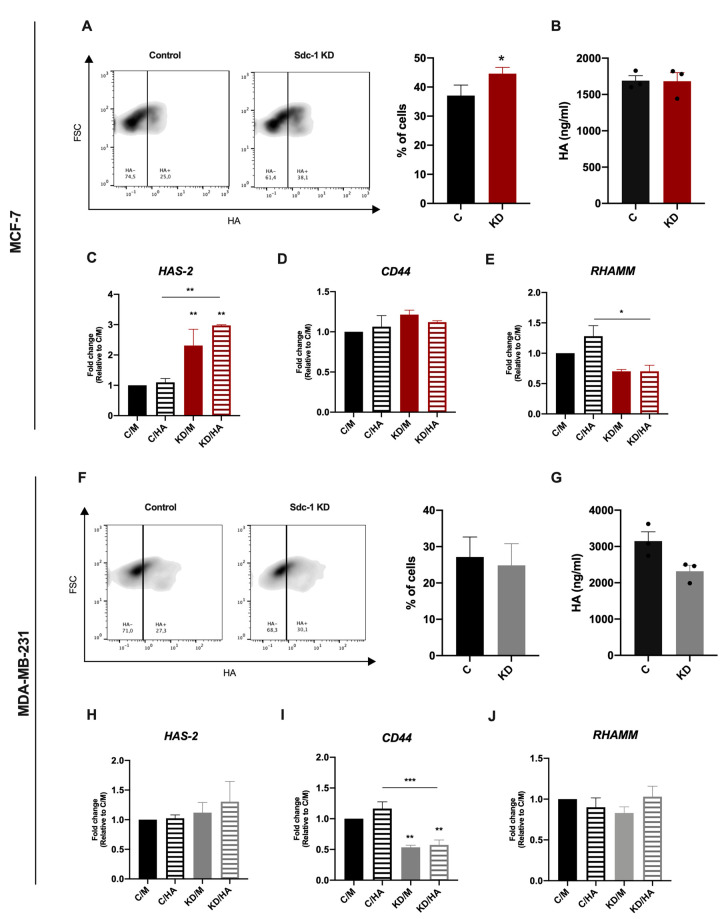
Sdc-1 KD increases HA binding and HAS-2 expression in MCF-7 cells, but not in the more aggressive MDA-MB-231 cells. mRNA levels of the HA receptors RHAMM and CD44 are diminished in Sdc-1-depleted MCF-7 and MDA-MB-231 cells, respectively. (**A**,**F**) Percentage of cells binding HA as analyzed by flow cytometry with HA-FITC staining after Sdc-1 depletion (*n* = 4 MDA-MB-231, *n* = 5 MCF-7). Representative density plots are shown. (**B,G**) HA measured in culture supernatants 48 hs post-transfection by ELISA-like assay (*n* = 3 MCF-7, *n* = 3 MDA-MB-231). (**C**–**E**, **H**–**J**) MCF-7 and MDA-MB-231 cells were transfected with siRNA and treated with 50 μg/mL HA for 24 h. HAS-2 (**C**,**H**), CD44 (**D**,**I**) and RHAMM (**E**,**J**) expression was determined by RT qPCR, normalized to β-ACTIN or GAPDH, and expressed as fold of C/M (*n* = 3 MCF-7, *n* = 3 MDA-MB-231). Data represent the mean ± SEM. Asterisks on the top of the bars indicate a significant difference between C/M and all other conditions. The line over the bars indicates a significant difference between C/HA and KD/M and KD/HA (* *p* < 0.05, ** *p* < 0.01, *** *p* < 0.001). C/M: control; C/HA: Control plus hyaluronic acid treatment; KD/M: Sdc-1 knockdown; KD/HA: Sdc-1 knockdown plus hyaluronic acid treatment.

To evaluate the impact of Sdc-1 downregulation on HA synthesis and function, we silenced Sdc-1 employing siRNA knockdown (KD) in the breast cancer cell lines MCF-7 (ER+, luminal subtype) and MDA-MB-231 (triple-negative, basal subtype). Successful Sdc-1 silencing under basal conditions and under HA treatment was confirmed by RT-qPCR (Supplementary Figure S1A). Previous studies had revealed that Sdc-1 siRNA knockdown is stable for at least 10 days, and that knockdown at the mRNA level results in a corresponding reduction at the protein level [17,18]. We confirmed our previous findings at the protein level for MDA-MB-231 cells (Supplementary Figure S1B). Exogenous HA addition did not alter Sdc-1 mRNA levels after 24 h of treatment. To study the impact of Sdc-1 depletion on HA metabolism, we analyzed the expression of HAS-2, one of the main enzymes involved in HA synthesis, by RT-qPCR. The soluble HA present in culture supernatants was tested by an ELISA-like assay, and we determined the HA binding ability of control and Sdc-1 KD cells by flow cytometry. In the less aggressive MCF-7 cells, we observed that Sdc-1 KD induced a slight but significant increase in the number of cells able to bind HA compared to control cells (Figure 1A). Sdc-1 KD did not affect the amounts of soluble HA (Figure 1B), but it increased the expression of HAS-2 after 48 h (Figure 1C). In the triple-negative and metastatic MDA-MB-231 cells, Sdc-1 KD resulted in a contrasting phenotype. No changes were observed in HAS-2 expression (Figure 1H) and a non-significant, slightly diminished HA release was observed (Figure 1G) 48 h after Sdc-1 downregulation. Besides, no changes in the HA binding ability were observed concerning control cells (Figure 1F). To study if the HA receptors CD44 and RHAMM were modulated when Sdc-1 was depleted, we analyzed their expression by RT-qPCR 48 h post-transfection under basal conditions. Additionally, we analyzed their expression under HA treatment to evaluate if the Sdc-1 depletion affects the HA modulation on its receptors. In Sdc-1-depleted MCF-7 cells, CD44 expression was not modulated, but RHAMM expression was significantly reduced, with HA with respect to control cells plus HA (Figure 1D,E). In contrast, Sdc-1 KD MDA-MB-231 cells showed significant downregulation in CD44 mRNA levels, with or without HA, for control cells with or without HA, while RHAMM expression was not modulated (Figure 1I,J).

### 2.2. Sdc-1 Depletion Inhibits Breast Cancer Stem Cell Properties in A Complex Interplay of EMT Transcription Factors and Wnt Signaling That Is Partially Modulated by HA

**Figure 2:**
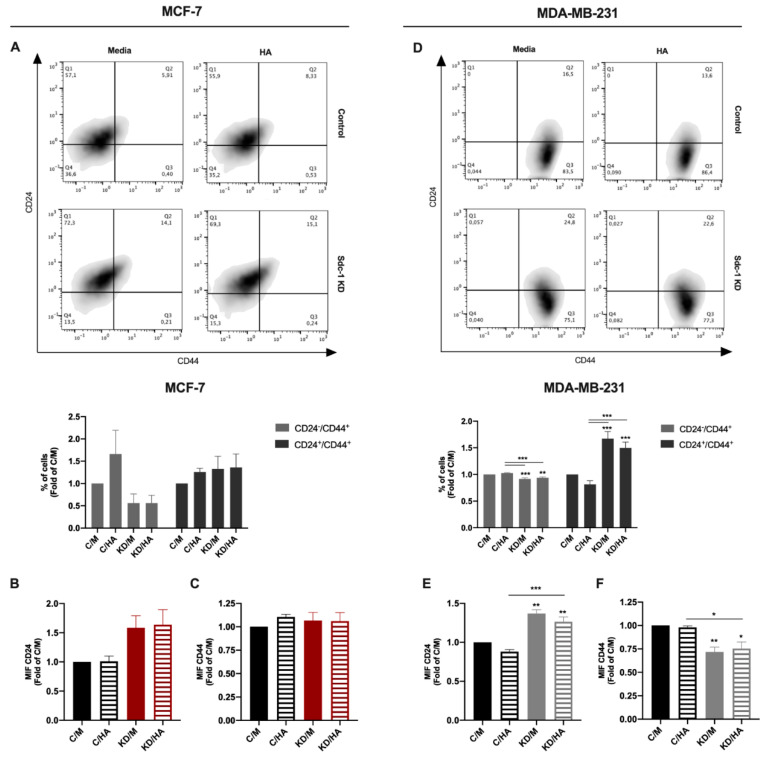
Depletion of Sdc-1 has a negative effect on the stem cell phenotype (CD44^+^/CD24^-^/low) of MCF-7 and MDA-MB-231 cells. (**A**,**D**) The percentages of CD24-/low/CD44^-^/low, CD24^-^/low/CD44high, CD24high/CD44^-^/low and CD24high/CD44high populations were measured by flow cytometry post-transfection and 96 h after 50 μg/mL HA treatment. Representative density plots are shown. (**B**–**C**,**E**–**F**) Mean intensity of fluorescence (MIF) of CD24 (**B**,**E**) and CD44 (**C**,**F**) was determined by flow cytometry and plotted as a fold of C/M (*n* = 5 MCF-7, *n* = 3 MDA-MB-231). Data represent the mean ± SEM. Asterisks on the top of the bars indicate a significant difference between C/M and all other conditions. The line over the bars indicates a significant difference between C/HA and KD/M and KD/HA (* *p* < 0.05, ** *p* < 0.01, *** *p* < 0.001). C/M: control; C/HA: control plus hyaluronic acid treatment; KD/M: Sdc-1 knockdown; KD/HA: Sdc-1 knockdown plus hyaluronic acid treatment.

A high expression of the cell surface marker and HA receptor CD44 combined with a low expression of CD24 (CD44^+^/CD24^−/low^) marks a breast cancer cell population with a CSC phenotype [29]. Since we previously showed that Sdc-1 KD had a negative effect on the CSC phenotype of triple-negative breast cancer cells [8,16], we explored the impact of Sdc-1 KD and HA treatment on this tumoral feature. Our results show that Sdc-1 KD had a similar effect in MCF-7 and MDA-MB-231 cells, reducing the CD44^+^/CD24^-/low^ and increasing the CD44^+^/CD24^+^ population concerning control cells; however, this difference was statistically significant only in MDA-MB-231 cells (Figure 2A,D). This effect was accompanied by a trend of an increase in the mean intensity of fluorescence of CD24 as a common characteristic in both cell lines, although CD44 cell surface expression was diminished only in MDA-MB-231 cells (Figure 2B,C,E,F). While no clear effect of HA was observed at the time point studied, the exogenous addition in control cells showed a trend for the reduction in the percentage of the CD24^+^/CD44^+^ population in MDA-MB-231 cells (Figure 2D).

**Figure 3:**
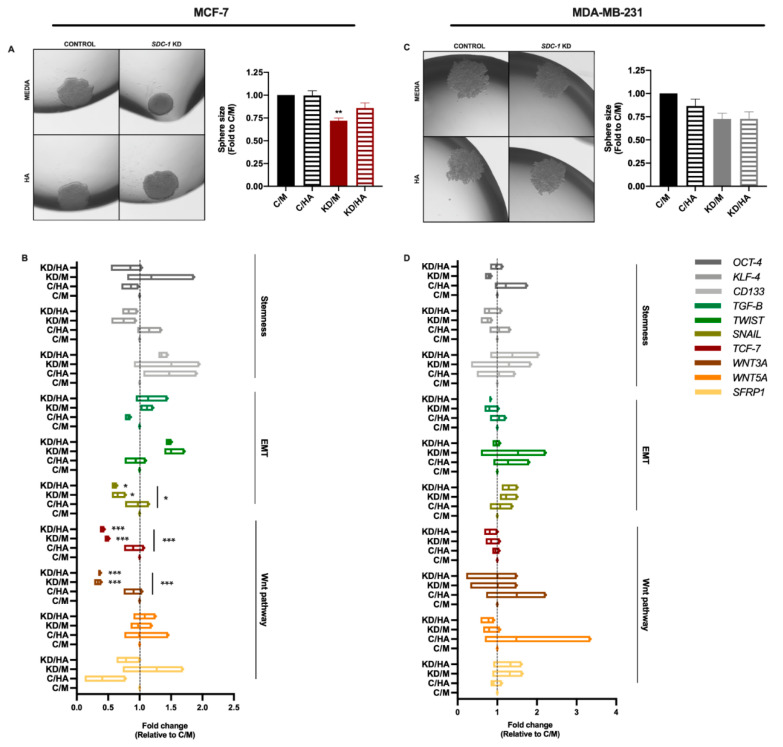
Sdc-1 KD reduces mammosphere size and downregulates EMT- and Wnt-related genes in MCF-7 cells. (**A**,**C**) Mammosphere size was determined by hanging drop assay and shown as a fold of C/M (*n* = 3). Representative mammospheres are shown. Data represent the mean ± SEM. (**B**,**D**) Expression of cancer stem cell-, EMT- and Wnt pathway-associated genes was analyzed post-transfection and 24 h after 50 μg/mL HA treatment by RT-qPCR, normalized to *β-ACTIN* expression, and shown as fold of C/M (*n* = 3 MCF-7, *n* = 4 MDA-MB-231). Data are presented as box and violin plots and means are represented with a line. Boxes with asterisks represent comparisons with statistically significant differences (* *p* < 0.05, ** *p* < 0.01, *** *p* < 0.001). C/M: control; C/HA: control plus hyaluronic acid treatment; KD/M: Sdc-1 knockdown; KD/HA: Sdc-1 knockdown plus hyaluronic acid treatment.

To investigate if the observed reduction in CSCs surface markers had an impact on tumorigenic capacity, we performed a 3D mammosphere formation assay. We detected a smaller size of mammospheres in both cell lines when Sdc-1 was depleted, being significant in MCF-7 cells (Figure 3A,C). The Sdc-1 KD-induced reduction was lost with the exogenous addition of HA in MCF-7 cells (Figure 3A). Furthermore, to determine if Sdc-1 KD and HA treatment modulate cell renewal and stemness-associated genes, we analyzed KLF-4, OCT-4, CD133, SOX2, and NANOG expression levels by RT-qPCR. KLF-4 expression showed a similar signature in both MCF-7 and MDA-MB-231 cells, with a trend of decrease when Sdc-1 was depleted, being significant at the protein level in MDA-MB-231 cells (Supplementary Figure S2A). Contrasting trends were found in MCF-7 and MDA-MB-231 cells regarding OCT-4 expression, and no clear effect was found for CD133 in these cell lines. We found no significant difference in NANOG and SOX2 expression in both cell lines after the treatments (data no shown).

Given the strong co-relation between the EMT process and the Wnt pathway regarding the CSC phenotype, we also analyzed the expression levels of the EMT-related genes *TGF-β*, *TWIST*, and *SNAIL,* by RT-qPCR. Moreover, for the Wnt pathway, we analyzed the gene expression of WNT3A and WNT5A, the negative regulator SFRP1 and the Wnt-dependent transcription factor TCF-7L1 (Figure 3B,D). We observed a strong negative effect of Sdc-1 KD on the expression of the canonical Wnt pathway ligand *WNT3A* and the transcription factor *TCF-7L1* in MCF-7 cells, but not in MDA-MB-231 cells. A trend of an increase in the Wnt inhibitor *SFRP1* was observed in Sdc-1-deficient cells of both cell lines; however, this increase was partially reduced by HA addition only in MCF-7 cells. In previous work of our group, it was demonstrated that low molecular weight (LMW) HA treatment increased β-catenin protein levels in MDA-MB-231 cells [3]. In accordance with this result, we observed a trend of an increase in Wnt ligands expression in the more aggressive MDA-MB-231 cell line, although it did not modify the Sdc-1 KD phenotype. HA did not modulate *WNT3A* and *TCF-7L1* expression in MCF-7, although a trend of a reduction in *SFRP1* expression was observed. Regarding EMT transcription factors, Sdc-1 KD reduced *SNAIL* expression levels, which, together with Wnt pathway downregulation, could account for the reduced CSC phenotype of Sdc-1-deficient cells. In MDA-MB-231 cells, TWIST showed a similar pattern of expression when compared to MCF-7 cells while SNAIL and TGF-β had an opposite one. These results suggest that a complex balance of these factors could influence both CSC phenotype acquisition mediated by Sdc-1 and/or HA.

### 2.3. Sdc-1 KD Exerts Different Effects on Cell Cycle Progression and Apoptosis in MCF-7 and MDA-MB-231 Cells with HA-Dependent Modulation of BAD Expression

**Figure 4:**
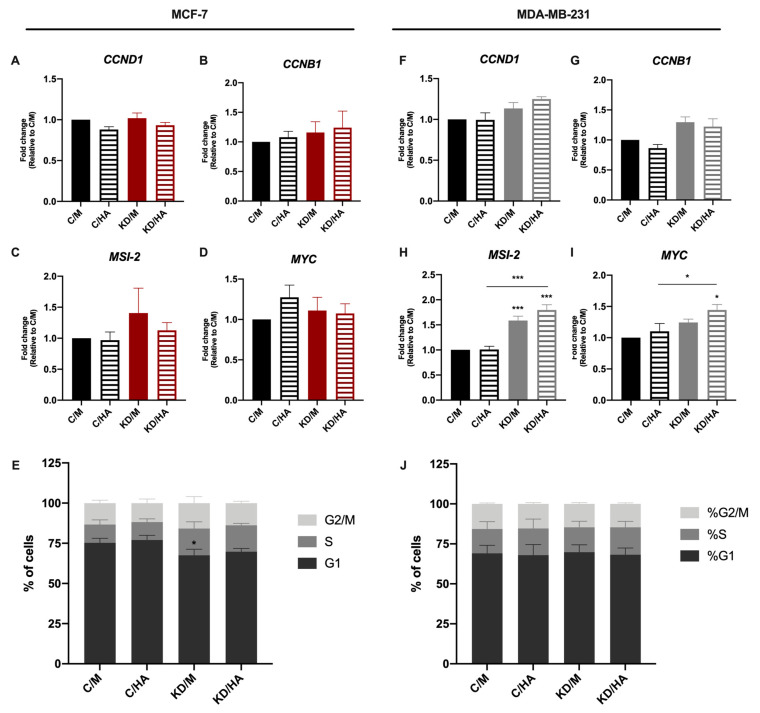
Sdc-1 depletion promotes cell cycle progression in MCF-7 cells and increases MSI-2 and MYC expression in MDA-MB-231. (**A**–**D**,**F**–**I**) Expression of *CCND1* (**A**,**F**), *CCNB1* (**B**,**G**), *MSI2* (**C**,**H**), and *MYC* (**D**,**I**) was analyzed post-transfection and 24 h after 50 μg/mL HA treatment by RT-qPCR, normalized to *β-ACTIN* expression, and shown as fold of C/M (*n* = 3 MCF-7, *n* = 4 MDA MB-231). Data represent the mean ± SEM. (**E**,**J**) Cell cycle phase composition was determined by DAPI staining and flow cytometry after Sdc-1 depletion and 96 h of 50 μg/mL HA treatment (*n* = 3 MCF-7, *n* = 4 MDA-MB-231). Asterisks on the top of the bars indicate a significant difference between C/M and all other conditions. The line over the bars indicates a significant difference between C/HA and KD/M and KD/HA (* *p* < 0.05, *** *p* < 0.001). C/M: control; C/HA: control plus hyaluronic acid treatment; KD/M: Sdc-1 knockdown; KD/HA: Sdc-1 knockdown plus hyaluronic acid treatment.

Since cell cycle and apoptosis are important processes related to tumorigenesis and cancer progression [26,27], we analyzed the expression of cell cycle-related genes and apoptosis by flow cytometry in both cell lines after the different treatments. First, we studied the mRNA levels of the cell cycle-associated genes CYCLIN D1 (CCND1), CYCLIN B1 (CCNDB1), MSI-2, and MYC 48 h post-transfection (24 h after HA treatment), and we observed no modifications in their expression in MCF-7 cells under the experimental conditions described (Figure 4A–D,F–I). However, a significant increase in *MSI-2* expression was detected in Sdc-1-depleted MDA-MB-231 cells for control cells, and a significant increase was observed when Sdc-1 KD was combined with exogenous HA treatment for *MYC* mRNA. To determine whether this change in gene expression had an impact on cell cycle progression, we analyzed the phase composition after Sdc-1 depletion and HA treatment. We observed that MCF-7 cells had a higher proportion of cells in the S phase in detriment of the G0/G1 phase than control cells, an effect that was independent of HA treatment. However, MDA-MB-231 did not show changes in their cell cycle phase composition (Figure 4E,J).

**Figure 5:**
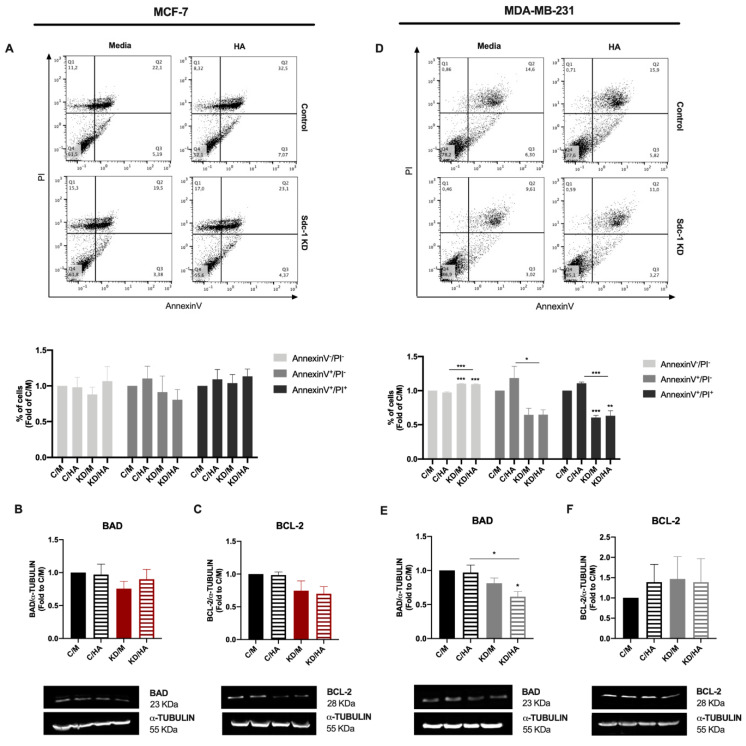
Sdc-1 deficient MDA-MB-231 cells have an anti-apoptotic profile in BAD and BCL-2 protein expression. (**A,D**) Apoptosis was determined after Sdc-1 depletion and 96 h of 50 μg/mL HA treatment by annexin V and PI staining by flow cytometry, and the percentage of viable (annexin V^−^/PI^−^), early apoptotic (annexin V^+^/PI^−^), and late apoptotic (annexin V^+^/PI^+^) cells are plotted as fold of C/M (*n* = 5 MCF-7, *n* = 3 MDA-MB-231). Representative dot plots are shown. (**B**–**C**,**E**–**F**) Pro-apoptotic BAD (**B**,**E**) and anti-apoptotic BCL-2 (**C,F**) protein levels were analyzed after Sdc-1 depletion and 48 h of 50 μg/mL HA treatment by Western blot, normalized to α-TUBULIN levels, and plotted as fold of C/M (*n* = 4 MCF-7, *n* = 4 MDA-MB-231). Representative bands are shown. Data represent mean ± SEM. Asterisks on the top of the bars mean a significant difference between C/M and all the conditions. The line over the bars indicates a significant difference between C/HA and KD/M and KD/HA (* *p* < 0.05, ** *p* < 0.01, *** *p* < 0.001). C/M: control; C/HA: control plus hyaluronic acid treatment; KD/M: Sdc-1 knockdown; KD/HA: Sdc-1 knockdown plus hyaluronic acid treatment.

Regarding cell death, we analyzed the percentage of apoptotic cells by flow cytometry determined by annexin V and PI staining, as markers of apoptosis or necrosis in both cell lines (Figure 5A,D). We observed that Sdc-1 depletion did not affect the levels of apoptotic or necrotic cells in the MCF-7 cell line (Figure 5A). However, a significant reduction in early (annexin V^+^/PI^-^) and late (annexin V^+^/PI^+^) apoptotic cells with a concomitant increase in viable cells was observed in MDA-MB-231 cells after the depletion of Sdc-1, with or without the addition of HA, with respect to control cells treated with or without HA (Figure 5D). Besides, we studied the protein levels of the BCL-2 family members BAD (pro-apoptotic) and BCL-2 (anti-apoptotic) 72 h post-transfection and 48 h after HA treatment (Figure 5B,C,E,F). MCF-7 cells presented no differences in BAD or BCL-2 levels under the conditions studied (Figure 5B,C). An opposite pattern was found for MDA-MB-231 cells, since the downregulation of BAD expression was found when Sdc-1 was depleted in addition to HA treatment (Figure 5E). No effects were observed regarding BCL-2 protein levels (Figure 5F).

### 2.4. Syndecan-1 KD Downregulates Angiogenic Factor and miR-10b Expression in Tumor Cells, while HA Addition Exerts Opposite Effects on IL-8 mRNA Levels in MCF-7 and MDA-MB-231 Cells

**Figure 6:**
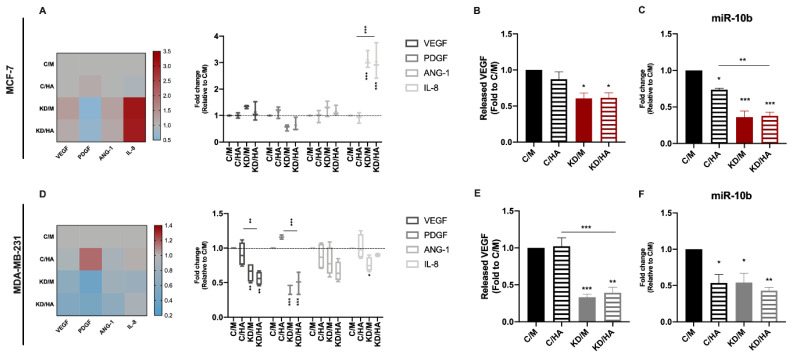
Sdc-1 KD exerts a negative impact on the production of the angiogenic factors VEGF, PDGF and IL-8 as on miR10-b expression in MDA-MB-231 cells, whereas a milder effect was observed in MCF-7 cells. (**A**,**D**) Expression of the angiogenic factors *VEGF, ANG-1, IL-8,* and *PDGF* was analyzed after Sdc-1 depletion and 24 h of 50 μg/mL HA treatment by RT-qPCR, normalized to *β-ACTIN* or *GAPDH* expression, and shown as fold of C/M (*n* = 3 MCF-7, *n* = 4 MDA-MB-231). Data are presented as a min-to-max box and whiskers plot and means are represented with a line symbol. Additionally, heat-maps representing means are shown. (**B**,**E**) VEGF released to culture supernatants was measured 48 h post-transfection and 24 h after 50 μg/m HA treatment by ELISA and plotted as a fold of C/M (*n* = 3 MCF-7, *n* = 3 MDA-MB-231). (**C**,**F**) *miR10b* expression was analyzed after Sdc-1 depletion and 24 h of 50 μg/mL HA treatment by RT-qPCR, normalized to the constitutive microRNA *RNU44,* and shown as a fold of C/M (*n* = 3 MCF-7, *n* = 3 MDA-MB-231). Data represent the mean ± SEM. Asterisks on the top of the bars mean a significant difference between C/M and all the conditions. The line over the bars means a significant difference between C/HA and KD/M and KD/HA (* *p* < 0.05, ** *p* < 0.01, *** *p* < 0.001). C/M: control; C/HA: control plus hyaluronic acid treatment; KD/M: Sdc-1 knockdown; KD/HA: Sdc-1 knockdown plus hyaluronic acid treatment.

Angiogenesis is essential for the growth and dissemination of tumor cells and it is well known that HA modulates the angiogenic process in a size-dependent manner [30]. Moreover, we have previously identified Sdc-1 as part of a pro-angiogenic signature in early breast cancer [11]. To study a possible cooperative effect of Sdc-1 and HA in breast cancer angiogenesis, we analyzed the expression of the angiogenesis-related genes *VEGF*, *PDGF*, *ANG-1*, and *IL-8* by RT-qPCR (Figure 6A,D). *VEGF*, *PDGF*, and *IL-8* were downregulated in the MDA-MB-231 cell line after Sdc-1 depletion regarding control. Moreover, a reduction in *PDGF* and *VEGF* expression was observed in the presence or absence of exogenous HA. However, *IL-8* reduction was impaired when HA was added. In MCF-7 cells, no significant differences were observed for *VEGF*, *PDGF,* or *ANG-1* but a strong increase in *IL-8* expression was found, contrary to what was observed in the MDA-MB-231 cell line. We also studied secreted levels of VEGF and bFGF (as a major angiogenic factor in tumors), in culture supernatants of control cells and cells transfected with Sdc-1 siRNA for 48 h and treated with HA for 24 h (Figure 6B,E). A decrease in VEGF release was found in both cell lines when Sdc-1 was depleted, but no effect was found for HA addition. The bFGF levels were below the reference so they could not be quantified.

Furthermore, a positive correlation was found between miR10b and angiogenesis in node-negative breast tumors [31], and since miR-10b regulates Sdc-1 expression in an estrogen-receptor-dependent manner [17,32], and since a link between HA-induced CD44 activation and miR-10b expression in MDA-MB-231 cells exists [33] we decided to analyze its expression levels by RT-qPCR (Figure 6C,F). We observed that miR-10b was downregulated regarding control in all the conditions studied in both cell lines. Altogether, our results suggest that in tumor cells, Sdc-1 KD decreases the expression of angiogenic factors, in a mechanism that could involve several pro-angiogenic molecules such as VEGF, PDGF, IL-8, and miR10b.

### 2.5. A Differential Kinase Activation Signature Is Associated with Sdc-1 Depletion in MDA-MB-231 and MCF-7 Cells

**Figure 7:**
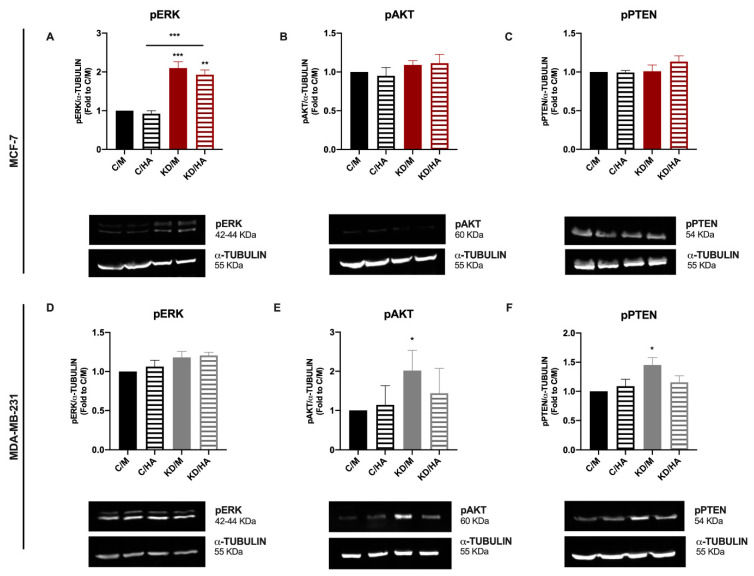
A differential kinase activation signature is observed in MDA-MB-231 and MCF-7 cells when Sdc-1 is depleted, increasing the activation state of PI3K and MAPK signaling pathways, respectively. (**A**–**F**) Phosphorylated forms of *p42/44 ERK (Thr202/Tyr204)* (**A**,**D**) *PTEN (Ser380)* (**B**,**E**) and *AKT (Ser473)* (**C**,**F**) were analyzed after Sdc-1 depletion and 48 h of 50 μg/mL HA treatment, detected by Western blot, normalized to *α-TUBULIN* levels and plotted as fold of C/M (n = 3 MCF-7, n = 4 MDA-MB-231). Representative bands are shown. Data represent the mean ± SEM. Asterisks on the top of the bars mean a significant difference between C/M and all the conditions. The line over the bars means a significant difference between C/HA and KD/M and KD/HA (* *p* < 0.05, ** *p* < 0.01, *** *p* < 0.001). C/M: control; C/HA: control plus hyaluronic acid treatment; KD/M: Sdc-1 knockdown; KD/HA: Sdc-1 knockdown plus hyaluronic acid treatment.

To understand which molecular signals are triggered in MCF-7 and MDA-MB-231 after Sdc-1 depletion could account for the observed effects on the studied processes, we analyzed the activation state of MAPK and PI3K/AKT signaling by Western blotting under our experimental conditions. We observed an increased expression of pERK in Sdc-1 deficient MCF-7 cells, whereas no significant modulation was observed in MDA-MB-231 cells (Figure 7A,D). Regarding the PI3K/AKT signaling pathway, the levels of PTEN phosphorylated at Ser380 (inactive) and AKT phosphorylated at Ser473 (active) were analyzed. Although no differences were found in MCF-7 cells, in MDA-MB-231 cells, active AKT (phosphorylated) and inactive PTEN (phosphorylated) forms were enhanced when Sdc-1 was depleted with respect to control cells, and this effect was abrogated when exogenous HA was added (Figure 7B,C,E,F). These results indicate that the differential activation of these pro-tumoral pathways could be involved in the different effects observed for Sdc-1 depletion in MCF-7 and MDA-MB-231 cells.

### 2.6. STRING Analysis Reveals That Sdc-1 Interacts with Various Protein Targets Associated with HA Metabolism, Stemness and Development, Adhesion, Angiogenesis, Growth, and Different Signaling Pathways

**Figure 8:**
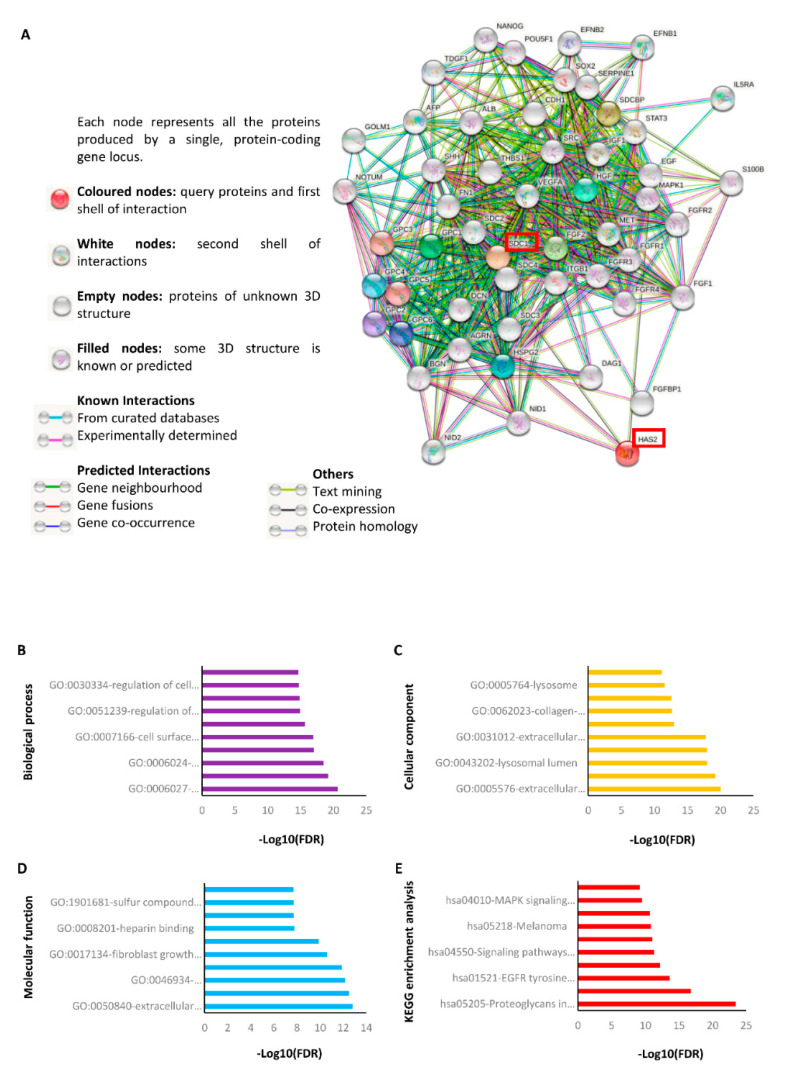
Protein–protein interaction network of SDC-1 and HA determined by STRING analysis. (**A**) Interaction network of SDC-1 and HA (red boxes) with proteins associated with angiogenesis, cell cycle, HA metabolism, stemness, EMT, and different signaling pathways. The analysis was performed using the STRING database (version 11) (https://string-db.org/cgi/network?taskId=bgCl7XhONyNJ&sessionId=bGLK0iM8ldY3, accessed on 7 January 2021). Gene ontology (GO) analysis of SDC-1 and HA (**B**) biological process, (**C**) cellular component, (**D**) molecular function, and (**E**) € KEGG pathway analysis. The ten most significant GO terms (*p* < 0.05) in cellular components (yellow), molecular functions (light blue), biological processes (purple), and KEGG pathways (red) branches are shown. The 10 most significant values were presented in (−log10) transformation. See Tables S1–S3 for further details.

To identify the protein interaction network associated with Sdc-1 and HA, we performed a silico analysis using the STRING (Search Tool for the Retrieval of Interacting Genes/Proteins) online tool [34]. The analysis showed that both Sdc-1 and HA synthase 2 (HAS2) directly interact with other proteoglycans including the syndecan (SDC2-4) and glypican (GPC1-6) family, agrin (AGRN), decorin (DCN), dermatan sulfate (BGN), perlecan (HSPG2), nidogens (NID1 and NID2), and other glycoproteins such as dystroglycan 1 (DAG1) (Figure 8A). Additionally, HAS2 interacts directly with epidermal growth factor (EGF) and fibronectin (FN1). On the other hand, Sdc-1 has close interaction with proteins associated with stemness and development (NANOG, OCT4 (POU5F1), SOX2, EFNB1, EFNB2, TDGF1, AFP), adhesion (SDCBP, ITGB1, THBS1, CDH1), growth (FGFR1-4), FGF2, EGF, HGF, VEGFA), signaling pathways (MAPK1, STAT3, MET, SRC, SHH, NOTUM), and other important proteins such as albumin (ALB), interleukin 5 receptor subunit alpha (IL5RA), Golgi membrane protein (GOLM1), serpin family E member 1 (SERPINE1), and S100 calcium binding protein B (S100B) (Figure 8A). We analyzed GO enrichment to evaluate the cellular component (yellow), molecular functions (light blue), and biological processes (purple) related to the proteins of interest. The ten most significant GO terms (*p* < 0.05) in each classification are presented in Figure 8B–D and Table S3. In the biological process group, the proteins were related to GAGs’ catabolic, metabolic, and biosynthetic processes, regulation of the developmental, multicellular organismal process, and cell migration (Figure 8B). Because of the enrichment of the cellular component, the proteins were associated with the extracellular region, Golgi, lysosomal, and vacuolar lumen, extracellular matrix, and space, as well as cell periphery and plasma membrane (Figure 8C). Finally, extracellular matrix, glycosaminoglycan, fibroblast growth factor, laminin, heparin, signaling receptor, and sulfur compound binding, and protein kinase activity were enriched in the molecular function group (Figure 8D). KEGG (Kyoto Encyclopedia of Genes and Genomes) pathway analysis (red) is shown in Figure 8E and Table S3. Remarkably, signaling pathways associated with regulating the pluripotency of stem cells, cell cycle, apoptosis, focal adhesion including PI3K-Akt, Rap1, EGFR, Ras, MAPK, and proteoglycans in cancer were enriched, as well as melanoma and bladder cancer (Figure 8E and Table S1). Moreover, we used STRING to predict any association between the analyzed targets based on PubMed co-citation analysis (Table S2) [34]. The analysis showed that Sdc-1 and HA and the proteins related to them were associated with “the role of PGs in breast cancer biology and translational medicine”, “Heparan sulphate and the art of cell regulation”, “Exploiting Heparan Sulfate Proteoglycans in Human Neurogenesis-Controlling Lineage Specification and Fate”, “Syndecans as modulators and potential pharmacological targets in cancer progression”, and “Proteoglycans in cancer biology, tumor microenvironment, and angiogenesis”, to cite some references (Table S2). In summary, this result confirms our experimental observation which indicates that Sdc-1 and HA are highly linked to proteins that participate in the activation of different signaling pathways playing an important role in cancer progression.

Finally, since our results show differences depending on the type of cell line (Luminal MCF-7 and triple-negative/basal MDA-MB-231), we analyzed the differential expression of Sdc-1 in non-tumor, primary tumors and metastases tissues from breast cancer patients. For this purpose, we use the online tool TNMplot (https://www.tnmplot.com, accessed on 21 May 2021) [35]. We observed that (1) Sdc-1 is more highly expressed in the primary and metastatic tumor tissues than in non-tumor samples and (2) Sdc-1 is expressed at higher levels in primary tissues than in metastatic sites (Supplementary Figure S3A). Subsequently, we evaluated the impact of Sdc-1 expression on the prognosis of the patients with breast cancer. We used the public online database Kmplot (https://kmplot.com/analysis/index.php?p=service&cancer=breast, accessed on 21 May 2021) [36]. We found that Sdc-1 expression correlates with worse relapse-free survival (RFS) in all the patients without any classification (*n* = 4929) (HR = 1.15 (1.04–1.27), *p* = 0.0073) (Supplementary Figure 3B). Interestingly, when the patients were filtered according to the expression of the estrogen receptor (ER) and the molecular subtypes Luminal A and basal, we found that the expression of Sdc-1 was associated with poor prognosis in the patients with ER-negative (ER-) (*n* = 1190) and basal (*n* = 846) tumors (HR = 1.3 (1.07–1.58), *p* = 0.0077; HR = 1.47 (1.18–1.85), *p* = 0.0007), respectively) (Supplementary Figure S3B). The expression of Sdc-1 had no association with RFS or ER-positive (ER+) (*n* = 2633) and Luminal A subtype (*n* = 2277) (Supplementary Figure S3B). These results support our in vitro experiments where Sdc-1 function appears to be tumor subtype-dependent.

## 3. Discussion

In the present study, we investigated the functional and regulatory relationship between the cell surface HSPG Sdc-1 and HA-related pathway constituents and biological/functional association in breast cancer employing widely used model cell lines representing luminal (MCF-7) and basal/triple-negative (MDA-MB-231) breast cancer. In agreement with previous findings in MDA-MB-231 cells with the reduced expression of the PG/GAG linker region biosynthetic enzyme ß4GALT7 [25], a reduced expression of Sdc-1 was associated with an upregulation of HAS2 in MCF-7 cells, while the expression of HA receptor, RHAMM, was downregulated. Notably, previous reports have shown that HA secretion is higher in MDA-MD-231 than MCF-7 and is in correlation with ER-negative status [37], but both cell lines express a similar level of mRNA HAS-2 and its gene expression is not associated with hormonal receptors’ ER/PR status [38,39]. Therefore, our results acquire significant weight to demonstrate that Sdc-1 and HA metabolism are functionally related, whereas Sdc-1 depletion also affected the HA signaling pathway in MDA-MB-231 cells, however, in a differential manner. When we analyzed the effect in the expression of HA, it is important to consider that MDA-MB-231 and MCF7 have different expressions of CD44 and RHAMM at the protein level, being lower in the last. Even more so, the interaction at the cell surface of these molecules is different in both cell lines [40]. This explains the results observed in Figure 1 regarding the differential expression of CD44 and RHAMM and HA binding capacity in both cell lines after Sdc-1 depletion. As a limitation of our study, we have to mention that only CD44, but not RHAMM levels were evaluated also at the protein level. While the Sdc-1-dependent changes were not sufficiently strong enough to translate into significant changes in HA secretion but significant changes were observed for binding, our data suggest that the depletion of Sdc-1 expression in MCF-7 cells could favor HA binding and probably its synthesis and modulate the capacity of HA to regulate the expression of RHAMM. In contrast, this mechanism was not observed in MDA-MB-231 cells, where Sdc-1 depletion mainly affected the HA receptor CD44 and had a negative effect on HA release, suggesting that differential signal pathways are modulated by HA according to the tumor phenotype. As reflected by our STRING analysis, Sdc-1 is a multifunctional signaling co-receptor, which affects numerous signaling pathways by enhancing interactions of a multitude of ligands with their receptors [41]. As a consequence, the actions of Sdc-1 are often contextual, as they depend on the configuration of Sdc-1-dependent signaling receptors in a given cell type. Our data confirm this concept, as a differential activation of signaling pathways was observed in our model cell lines. In MCF-7 cells, Sdc-1 depletion resulted in an upregulation of activated Erk under our experimental conditions, whereas Sdc-1-depleted MDA-MB-231 cells showed the increased activation of AKT and a reduction in PTEN activation (as indicated by the presence of higher levels of phosphorylated PTEN) [18]. These data conformed with the literature, as PTEN is an important negative regulator of PI3K/AKT signaling, acting as a tumor suppressor [42] Initially, the upregulation of Erk phosphorylation in MCF-7 cells appears surprising, as we previously showed that Sdc-1 depletion reduces Erk activation of serum-starved MCF-7 cells in response to bFGF stimulation. Under our experimental conditions, alternative pathways, such as integrin-dependent signaling [15], may account for this observation. Furthermore, our present findings are consistent with the upregulation of Erk activation that has been observed in the mammary glands of Sdc-1 knockout mice [36]. While exogenous HA did not affect Sdc-1-dependent Erk signaling in MCF-7 cells, it reduced the Sdc-1-dependent impact on AKT and PTEN signaling in MDA-MB-231 cells, providing an important functional link between these signaling constituents. The activation of different cell signals of HA by CD44, as well as by RHAMM in breast cancer, is well documented [43]. It has been observed that these receptors form a complex activating the ERK proteins in breast cancer cells. Additionally, this activation is associated with motility and invasion capacity of these cells, where the functional impact is stronger observed in MDA-MB-231 than in MCF-7 cell lines. On the other hand, like our results, differential effects of HA in these cells could be related to the differential isoform expression of its receptors and basal levels of ERK phosphorylation [40]. Similarly, when analyzing the PI3K/Akt signal, it is important to remember that HA by its receptors promotes PI 3-kinase signaling in a tumor cell-specific manner. Importantly, Bourguignon et al. described that this signal-mediated breast tumor progression by the stimulation of cytokine productions, tumor cell growth, survival, and invasion [44]. Besides, it has been demonstrated that hyaluronan synthesis by a clone of MDA-MB-231 that can metastasize to the bone, required for the EGF-mediated activation of focal adhesion kinase/PI3K/Akt signaling associated with the invasive phenotype [45]. However, in this analysis, is important to remake that the HA signal is dependent on multiples mechanisms [46] where molecules expressed in the plasma membrane could affect direct or indirect crosslinking of HA receptors and therefore downstream signal activation, because of our results one of them could be Sdc-1. Our finding of a reduced expression of the miRNA miR-10b both in Sdc-1-depleted and HA-treated breast cancer cells may serve to explain this effect in MDA-MB-231 cells, as it was recently shown that miR-10b expression in breast cancer stem cells supports self-renewal through negative PTEN regulation and sustained AKT activation [47]. In line with this observation, our STRING analysis indicates that the interaction between Sdc-1 and PTEN may indeed be indirect and may involve CD44 and AKT as linking factors. Previous work by the Bourguignon group had indicated a link between HA-induced CD44 activation and miR-10b expression in MDA-MB-231 cells, involving c-Src and Twist [33]. Following short-term stimulation (2 h) with 50 µg/mL 4–5 × 10^5^ Da HA, they showed the induction of miR-10b expression using RNAse protection assays. In our hands, long-term stimulation (24 h) with the same concentration of a slightly smaller (1–3 × 10^5^ Da) HA resulted in a downregulation of miR-10b expression, as detected by qPCR. While these data underline a role for HA in regulating miR-10b expression, they suggest that the timing of HA application has an important regulatory impact. While we previously showed that miR-10b is expressed depending on the estrogen receptor status in breast cancer cells [32], Sdc-1-dependent miR-10b regulation was observed in both ER-positive and -negative model cell lines in our study.

On the other hand, the observed Sdc-1 and HA-dependent changes in signaling were associated with changes in gene expression that could be linked to several phenotypes relevant to breast cancer progression. In line with previous studies of our laboratory, Sdc-1 depletion affected the breast cancer stem cell phenotype, associated with therapeutic resistance [8,16]. The present study confirms and extends these findings by demonstrating that Sdc-1 depletion inhibits MCF-7 sphere formation in the hanging drop assay, a phenotype that could be partially compensated by exogenous addition of HA, suggesting a potential contribution of HA-dependent pathways to this phenotype. Moreover, we could demonstrate a significant negative impact of Sdc-1 depletion on CD44 expression and an increase in CD24 + cells, and therefore a loss on the CD44^+^/CD24^−/low^ breast CSC phenotype of MDA-MB-231 cells. This is following our previous observation of a co-regulation of CD44 and Sdc-1 in clinical samples of inflammatory breast cancer [8]. Since we observed a diminished of the number of CD24/cells, represented in Figure 2 as MIF of CD24 (fold of C/M), we could speculate on a possible mechanism associated to Sdc-1. Recently, a role for Sdc-1 in the recycling of receptors has been described [48]; thus, the loss of Sdc-1 by its depletion in our cellular model might have affected the internalization of CD24, hence increasing its expression at the cell membrane.

We also observed that the impact on the stem cell phenotype was associated with transcriptional changes in markers relevant to stem cell progression, notably the downregulation of the transcription factors OCT-4, SOX2, and KLF4, one of the ‘Yamanaka-factors’ required for the reprogramming of differentiated cells into an induced pluripotent stem cell-like state [49], which appeared to be due to Sdc-1-dependent transcriptional mechanisms in MCF-7 and posttranscriptional regulation in MDA-MB-231 cells. Overall, our STRING analysis suggests that this regulation occurs indirectly, as OCT4 and SOX2 were neither in the first interaction shell for Sdc-1. Additionally, the KEGG enrichment analysis showed that Sdc-1 and its related proteins participate in the regulation of pluripotency factors. We observed the downregulation of the Wnt pathway ligand Wnt-3A and the Wnt-dependent transcription factor TCF7L1 in Sdc-1-depleted MCF-7 cells, which is in accordance with our previous observation of a downregulation of the Wnt-coreceptor LRP6 in Sdc-1-depleted breast cancer cells [16]. We have recently demonstrated that siRNA knockdown of the RNA binding protein, which is also a stem cell marker, Musashi (Msi) expression results in a loss of CSC features and decreases the radioresistance capacity of the triple-negative breast cancer cells. However, invasion, growth and cell motility were enhanced upon Msi knockdown [50], generating a phenocopy of Sdc-1 depletion in MDA-MB-231 cells and suggesting a functional interaction [17]. In contrast, in this study, in the triple-negative MDA-MB-231 cells, Msi2 was upregulated upon Sdc-1 depletion, suggesting a feedback loop between Sdc-1 and Msi. Finally, the expression of the EMT-associated mesenchymal marker SNAIL was downregulated in Sdc-1-depleted MCF-7 cells. Interestingly, CDH1 (E-cadherin), which is a target of SNAIL, was also identified as a direct interaction partner of Sdc-1 in our STRING analysis. Overall, these data expand our previous findings of an Sdc-1-dependent modulation of a CSC phenotype in breast cancer cells via the IL6/STAT3-, Wnt- and Notch-pathways and suggest that Sdc-1 knockdown may induce differentiation of the CSC population, thus attenuating malignant properties. Interestingly, we observed in our STRING analysis that Wnt and STAT3 has also indirect but close interaction with Sdc-1 and HA. However, except for the attenuation of the effect of Sdc-1 depletion on MCF-7 sphere formation, we did not observe a modulation of the Sdc-1-dependent changes in CSC properties by the exogenous addition of HA under our experimental conditions. In our triple-negative cell line, the downregulation of CD44 may have negatively affected their response to HA and may have partially contributed to this observation. As we mentioned above, the biological effects of HA are dependent on multiples mechanics and context; one of these is the cellular microenvironment, where immune, fibroblast, endothelial and mesenchymal cells modulated HA synthesis, molecular weight, distribution, synthesis, or degradation. In this context, we have published that in breast cancer cell responses to drugs, the angiogenic action is associated with the presence of macrophages and endothelial cells [51,52]. Thus, further studies will be necessary to fully understand the association between HA and SDC-1, taking into account the tumor microenvironment. Altogether, these results indicate that Sdc-1 depletion has a negative impact on the CSC phenotype of both breast cancer cell lines, as we observed a reduction in CD44 expression in MDA-MB-231 cells. Although we observed an increase in CD24 in MCF-7, it does not mean that the stem population is decreasing, since we also observed a slight increase in CD44, but in neither case was it significant. This reduction in CD44 in the cell surface could explain why HA exogenous addition was not able to restore mammosphere growth when added to Sdc-1 KD cells in the more aggressive MDA-MB-231 cells.

It has been described that ECM components such as HA, collagen, and fibronectin can control cell cycle checkpoints through receptors, integrins, and syndecans [27]. Apart from the impact on the CSC phenotype, Sdc-1 depletion resulted in a change in cell cycle progression in MCF-7 cells, increasing the number of cells in the S-phase. Apart from the increased activation of Erk, the alterations in RHAMM expression could be associated with the cell cycle since it is known to interact both with Erk and bind to the mitotic spindle [40,53]. Notably, HA treatment abolished the impact of Sdc-1 depletion on the cell cycle, indicating a dependence of Sdc-1-functions on HA signaling. While we previously demonstrated a moderate effect of Sdc-1 on MDA-MB-231 cell viability [17], we did not observe any impact on cell cycle progression under our experimental conditions, suggesting subtype-specific effects on breast cancer cell proliferation. As the net growth of tumors depends both on cell proliferation and cell death [26,27], we determined the impact of Sdc-1 depletion and HA treatment on apoptosis and necrosis in our model cell lines. In contrast to a study on Sdc-1 and n-3 polyunsaturated fatty acid-related regulation of apoptosis [54,55], we did not observe any impact on apoptosis in MCF-7 cells. Differences in study methodology (annexin V vs. cleaved PARP/caspase apoptosis assays) may have accounted for this discrepancy. However, Sdc-1 depletion significantly decreased the number of both apoptotic and necrotic MDA-MB-231 cells. At the molecular level, these changes were accompanied by a consistent decrease in the expression of the pro-apoptotic factor BAD [56] in the presence of HA. The increased AKT activation under Sdc1 knockdown conditions could have further enhanced the anti-apoptotic effect through the downregulation of BAD. Moreover, the combination of Sdc-1 depletion and HA treatment resulted in a synergistic upregulation of cMyc expression, which may have further contributed to the anti-apoptotic effect [57]. While Sdc-1 depletion was sufficient to exert an anti-apoptotic function in MDA-MB-231 cells, our data indicate that HA promotes molecular mechanisms that strengthen this phenotype.

Finally, our study demonstrates an impact of Sdc-1 on the expression and release of angiogenic factors. Indeed, Sdc-1 is co-expressed with angiogenic factors in early stages of breast cancer [11], and studies in Sdc-1 knockout and overexpressing mice [58,59], and on proangiogenic integrins [60] have identified alterations in proteolysis, integrin activity, and integrin-dependent leukocyte recruitment as contributing mechanistic factors for a role of Sdc-1 in angiogenesis. Our STRING analysis supports this finding, demonstrating interactions of Sdc-1 with VEGF, integrins, other adhesion proteins, and MMPs. Consistently, we detected a decreased release of the key angiogenic cytokine VEGF into the culture media of both MCF-7 and MDA-MB-231 cells upon Sdc-1 knockdown. Regarding additional angiogenic factors, our qPCR analysis revealed a differential impact of Sdc-1 depletion in MDA-MB-231 and MCF-7 cells, with a reduction in VEGF, PDGF, and IL-8 levels in the basal cell line, and an upregulation of IL-8 in the luminal cell line, suggesting that Sdc-1-dependent VEGF regulation occurs in a posttranscriptional manner in MCF-7 cells. Finally, according to our and other results, the co-citation analysis done on the String platform showed that both Sdc-1 and HA as well as the proteins related to them participate in breast cancer biology, angiogenesis, and tumor microenvironment [61,62].

## 4. Conclusions

The present study highlights an important impact of Sdc-1 depletion on several protumorigenic breast cancer cell properties, including the CSC phenotype (associated with therapeutic resistance), cell cycle progression, apoptotic resistance, and expression of proangiogenic factors. Overall, these data point at an important role of Sdc-1 as a tumor suppressor, particularly in triple-negative breast cancer. By the pleiotropic role of cell surface HSPGs [41], the molecular pathways affected by Sdc-1 were regulated in a subtype-specific manner and included the modulation of Erk, Akt, and PTEN signaling, associated transcription factors (KLF4, TCF7L1), cytokines, and morphogens (VEGFA, Wnt) as well as post-transcriptional regulators and epigenetic factors (Msi2, miR-10b). While Sdc-1 depletion was associated with significant subtype-specific expression changes in HA-related factors (HAS2, CD44, RHAMM), our experiments employing the exogenous addition of HA suggest that HA has more of a modulating effect on Sdc-1-dependent processes in breast cancer, which partially acts synergistically. Overall, our data confirm a role for Sdc-1 and the HA pathway in breast cancer that together are playing an important role in tumor progression and therefore mark these molecules as attractive therapeutic targets in breast cancer.

## 5. Materials and Methods

### 5.1. Cell Culture

The human breast cancer cell lines MCF-7 and MDA-MB-231 were purchased from ATCC/LGC Promochem (Wesel, Germany). MCF-7 cells were cultured in RPMI-1640 containing 1% glutamine (cat. no. R8758), 10% fetal calf serum (FCS) (Biochrom GmbH, cat. no. S0615, Berlin, Germany), and 1% penicillin/streptomycin (cat. no. P4333) in a humidified atmosphere of 5% CO_2_ at 37 °C. MDA-MB-231 cells were cultured in Dulbecco’s Modified Eagle Medium (DMEM) (cat. no. D0819) containing 10% FCS, 1% glutamine, and 1% penicillin/streptomycin in a humidified atmosphere of 7.5% CO_2_ at 37 °C. All reagents except for FCS were purchased from Sigma-Aldrich Chemie GmbH, Taufkirchen, Germany.

### 5.2. siRNA Transfection

MCF-7 and MDA-MB-231 cell lines were cultured in growth media at a density of 2.5 × 10^5^, 2.0 × 10^5,^ or 1.75 × 10^5^ cells/well of a six-well plate for 24, 48, or 96 h of HA treatment, respectively. After 24 h, siRNA transfection was performed using Dharmafect reagent (Dharmacon™, cat. no. T-2001-03, Lafayette, CO, USA). Briefly, each well had 1 mL of the final volume consisting of 840 µL of Opti-MEM^®^ media (Gibco™, Thermo-scientific cat. no. 31985-070, Bremen, Germany), 80 µL of 20 nM Sdc-1 siRNA/Opti-MEM^®^ (Ambion^®^ life technologies, cat. no. s12634, Cambridgeshire, UK) or negative control siRNA (Ambion^®^ life technologies, cat. no. 4390844, Cambridgeshire, UK), and 80 µL of 2.5% Dharmafect/Opti-MEM^®^ solution. Cells were incubated for 24 h and then transfection media was replaced for growth media. The knockdown efficiency was confirmed by RT-PCR (Supplementary Figure S1).

### 5.3. HA Treatment

Recombinant LMW HA (1–3 × 10^5^ KDa CPN, Czech Republic) was kindly supplied by Farmatrade S.R.L (Villa Lynch, B.A., Argentina) and diluted in ultra-pure water (pyrogen and nucleases free) to a final concentration of 5 mg/mL. For HA treatment, growth media of both cell lines was replaced for 1% FCS media, and 50 μg/mL LMW-HA was added for 24, 48, or 96 h according to the experiment.

### 5.4. Total RNA Extraction and cDNA Synthesis

For RNA experiments, 2.5 × 10^5^ cells were cultured in a six-well plate and transfected with control or Sdc-1 siRNA as described before and treated with HA for 24 h before the collection of the samples. Total RNA extraction was performed with InnuPREP RNA mini kit (Analytikjena, cat. no. 845-KS-2040250, Jena, Germany) following the manufacturer’s instructions. Retro-transcription of mRNA (from 1 µg of RNA) and microRNA (from 0.1 µg of RNA) into cDNA was performed with the High-Capacity cDNA Reverse Transcription Kit (Applied Biosystems, cat. no. 4368814, Foster City, CA, USA) and TaqMan™ MicroRNA Reverse Transcription Kit (Applied Biosystems, cat. no. 4366596, Foster City, CA, USA), respectively. For microRNA transcription, sequence-specific probes were employed for the target microRNA *miR10b* (ID 002218) and the internal control *RNU44* (ID 001094) (Applied Biosystems).

### 5.5. Quantitative Real-Time PCR

qPCR for each gene was performed using Power SYBR™ Green PCR Master Mix Applied Biosystems, Thermo Fisher, cat. no. 4367659, Foster City, CA, USA) and Takyon™ No ROX Probe 2X MasterMix UNG (Eurogentec, cat. no. UF-NPMU-C0701, Seraing, Belgium). Gene expression was measured with the ABI 7300 Real-time PCR Detection System (Applied Biosystems, CA, USA). The ∆∆CT method was employed for the transcriptional analysis and target gene expression was normalized to *β-ACTIN* expression levels. *RHAMM, HAS-2,* and *PDGF* expression levels were quantified using SsoAdvanced Universal SYBR Green Supermix (1725271, Bio-Rad Laboratories, Hercules, CA, USA). Gene expression was measured with the CFX96 Touch Real-Time PCR Detection System (Bio-Rad, Laboratories, Hercules, CA, USA). The ∆∆CT method was employed for the transcriptional analysis and target gene expression was normalized to *GAPDH* expression levels. Primer and probe sequences were confirmed by NCBI BLAST analysis and are shown in the Supplementary Table S3.

### 5.6. Protein Extraction

For protein experiments, 2.0 × 10^5^ cells were cultured in six-well plates, transfected with control or Sdc-1 siRNA as described before, and treated with HA for 48 h. Samples were collected in 100 µL of 2× protein sample buffer Laemmli and sonicated with Ultrasonic Processor UP100H (Hielscher GmbH, Hamm) to prepare cell lysates.

### 5.7. SDS-Page and Immunoblotting

An amount of 15 µL of protein of each sample was separated on 10% SDS gels at 120-150V, and electro-transferred for 1 h at 16V into nitrocellulose membranes (Amersham, Pharmacia Biotech, cat. no. 10600008, Piscataway, NJ, USA). The unspecific binding blockade was performed with 5% (w/v) skimmed milk powder in TBS-T buffer for 1 h shaking at room temperature (RT). Membranes were incubated with primary antibodies in a 1:1000 or 1:5000 dilution in 5% BSA-TBS-T overnight at 4 °C. The HRP-conjugated secondary antibody was used at a final concentration of 1:5000 for 1 h at RT. Membranes were subjected to chemiluminescence ECL reaction with SuperSignal™ West Pico PLUS Chemiluminescent Substrate (Thermo Scientific™, cat. no. 34580, Foster City, CA, USA) in a FUSION SL (Vilber Lourmat, Marne-la-Vallée Cedex, France) device and the visualized bands were quantified with NIH ImageJ software. The primary and secondary antibodies used are listed in Supplementary Table S4.

### 5.8. Flow Cytometry

For flow cytometry, 1.75 × 10^5^ cells were cultured in a six-well plate and transfected with control or Sdc-1 siRNA and treated with HA as described before. When 96 h had passed after HA treatment, cells were collected with 2 mM EDTA (AppliChem, cat. no. 141669.1211, Darmstadt, Germany) in PBS–FBS 2%, washed twice with PBS, and stained for the following protocols.

#### 5.8.1. CD24/CD44 Population Analysis

For the analysis of the expression of cell surface breast CSC markers, cells were incubated with 10 μL of APC Mouse Anti-Human CD44 (BD Pharmingen™, cat. no. 559942, Franklin Lakes, NJ, USA), PE Mouse Anti-Human CD24 (BD Pharmingen™, cat. no. 555428, Franklin Lakes, NJ, USA) as with APC and PE isotype control antibodies for 20 min at RT in the dark. Stained cells were analyzed on a flow cytometer CyFlow space (Sysmex PARTEC GmbH, Kobe, Japan). Data analysis was performed with FloMax software (Partec) and representative plots were performed with FlowJo software (LLC).

#### 5.8.2. Cell Cycle Analysis

For DAPI staining of cells, pellets were resuspended in 1 mL of 4,6-diamidino-2-phenylindole (DAPI) (CyStain UV Ploidy, cat. no. 05-5001, Sysmex, Norderstedt, Germany) and after 5-min incubation at RT, cells were analyzed by flow cytometry on a flow cytometer CyFlow space (Sysmex PARTEC GmbH). Excitation was carried out with a 375-nm UV laser and fluorescence emission was measured at 455 nm in FL4. Data analysis was performed with FloMax software (Partec) and representative plots were performed with FlowJo software (LLC).

#### 5.8.3. Apoptosis Analysis

Cells were stained with the FITC Annexin V Apoptosis Detection Kit (BD Pharmingen™, cat. no. 556547, Franklin Lakes, NJ, USA). Briefly, cells were resuspended in 1 mL of 1X Binding Buffer and 100 µL of the cell suspension was transferred to another tube and 5 µL of annexinV-FITC antibody and 5 µL of propidium iodide (PI) were added to all samples except for the auto-fluorescence control. After 15 min of incubation at room temperature (RT) in the dark, labeled cells were measured on a flow cytometer CyFlow space (Sysmex PARTEC GmbH). Cells were excited with a 488 nm blue argon laser and signals were collected at 527 nm in FL1 and 665 nm in FL3. Data analysis was performed with FloMax software (Partec) and representative plots were performed with FlowJo software (LLC).

### 5.9. HA Binding Ability

To determine the HA binding ability of control and Sdc-1 KD MCF-7 and MDA-MB-231 cells, 1.75 × 10^5^ cells were cultured in a six-well plate and transfected with control or Sdc-1 siRNA as described before, and 5 days post-transfection were collected with 2mM EDTA in PBS–FBS 2%. Once centrifugated at 1500 rpm and washed, cells were resuspended in 100 µL of PBS–BSA 0.1% and 50 μg/mL of hyaluronic acid, bovine trachea, fluorescein-labeled (HA-FITC) (Calbiochem, Merck KGaA, cat. no. 385906, Darmstadt, Germany) and incubated for 1 h at 4 °C in the dark. The analysis was performed by flow cytometry on a flow cytometer CyFlow space (Sysmex PARTEC GmbH). Excitation was carried out with a 488 nm blue argon laser and signals were collected at 527 nm in FL1. Data analysis was performed with FloMax software (Partec) and representative plots were performed with FlowJo software (LLC).

### 5.10. HA Release

HA present in culture supernatants of Sdc-1 KD cells was measured after 48 h post-transfection using a commercial Hyaluronan Enzyme-Linked Immunosorbent Assay (HA ELISA) (Echelon Biosciences, cat. no. K-1200, Salt Lake City, UT, USA) according to the manufacturer instructions.

### 5.11. ELISA

The levels of VEGF and bFGF present in conditioned media of MCF-7 and MDA-MB-231 cells were measured after 24 h of HA treatment by Human VEGF DuoSet ELISA Kit (R&D Systems, cat. no. DY293B-05, Minneapolis, MN, USA) and Human FGF basic/FGF2/bFGF DuoSet ELISA (R&D Systems, cat. no. DY233, Minneapolis, MN, USA) according to the supplier’s instructions.

### 5.12. Hanging Drop Assay

For mammosphere formation in hanging drops, 2.5 × 10^5^ cells were cultured in a six-well plate and transfected with control or Sdc-1 siRNA. After 24 h post-transfection, cells were collected with Accutase^®^ solution (Cell Detachment Solution, w: 0.5 mM EDTA, w: Phenol red) (Sigma-Aldrich Chemie GmbH, cat. no. A6964, Taufkirchen, Germany) and centrifugated. A suspension of 400,000 cells/mL was prepared, and it was split into two tubes, where nuclease-free water (Sigma-Aldrich Chemie GmbH, cat. no LSKNF0500, Taufkirchen, Germany) or HA was added in a final concentration of 50 µg/mL. Then, 30 µL drops containing 12,000 cells were displayed on the upper side of Petri dishes, which were incubated for 6 days in a humidified atmosphere of 5 or 7.5% CO_2_ at 37 °C. Finally, Petri dishes were inverted and mammosphere pictures were taken with Zeiss Axiophot (Zeiss, Jena, Germany) bright-field microscope (magnification 10×). The mammosphere area was quantified with the NIH ImageJ program (U.S. National Institutes of Health, Bethesda, Maryland, USA).

### 5.13. STRING Protein-Protein Interaction Analysis

The online platform STRING (https://string-db.org/cgi/network?taskId=bgCl7XhONyNJ&sessionId=bGLK0iM8ldY3, accessed on 7 January 2021, https://string-db.org accessed on 28 April 2021) [34] was used to develop the in-silico protein interaction networks for Sdc-1 and HA. All interactions were predicted with a high confidence threshold of 0.700. Additionally, for the enrichment analysis, STRING uses three different databases: GO, Pfam (Protein families), and KEGG and performs a multiple testing correction separately within each functional classification framework (GO, KEGG, InterPro, etc.), according to Benjamini and Hochberg, to predict protein–protein interaction (PPI) networks [34].

### 5.14. TNM and Kaplan–Meier Plots

The TNMplot database (https://www.tnmplot.com/, accessed on 21 May 2021) [35] was set up using the RNA-seq from The Cancer Genome Atlas (TCGA) repository. The database contains 11,010 samples from TCGA: 394 metastatic, 9886 tumorous and 730 normal tissues. Comparison of the normal and the tumor primary samples was performed by the Mann–Whitney U test, and matched tissues with adjacent samples were compared using the Wilcoxon test. Normal, primary tumors and metastatic tissues gene comparison were also analyzed using Kruskal–Wallis test. The statistical significance cutoff was set at *p* < 0.01 [35]. For the survival analysis, the publicly available gene expression database Kaplan–Meier plotter (KM plotter) (https://kmplot.com/analysis/index.php?p=service&cancer=breast, accessed on 21 May 2021) was used [36]. The database was established using gene expression data and survival information downloaded from Gene Expression Omnibus (GEO). The patients were stratified by estrogen receptor (ER), as well as for the molecular classification Luminal A and basal. We analyzed the expression of Sdc-1 and visualized its correlation to survival by generating Kaplan–Meier survival plots. High and low expression of Sdc-1 were determined using the median as a cut-off. The Affymetrix probe set ID for Sdc-1 is 201286_at.

### 5.15. Statistical Analysis

For statistical analysis, 95% confidence intervals (CI) were determined by calculating arithmetic mean values and variance (standard deviation, SD) of at least three independent experiments. To evaluate whether differences between the values obtained were significant, the t Student’s test (*t*-test, Mann–Whitney) was used in the case of comparisons between two groups and one-way ANOVA with Tukey’s multiple comparisons test was used in the case of comparisons between more than two groups. The software Prism (GraphPad, San Diego, CA, USA) was used, considering a *p* value < 0.05 as statistically significant.

## Data Availability

All data presented in this study are available in the manuscript and Supplementary Files.

## Supplementary Materials

Supplementary materials can be found at https://www.mdpi.com/article/10.3390/ijms22115874/s1.

## References

[1] Theocharis A.D., Skandalis S.S., Gialeli C., Karamanos N.K. (2016). Extracellular matrix structure. Adv. Drug Deliv. Rev..

[2] Kang H., Wu Q., Sun A., Liu X., Fan Y., Deng X. (2018). Cancer cell glycocalyx and its significance in cancer progression. Int. J. Mol. Sci..

[3] Vitale D., Kumar Katakam S., Greve B., Jang B., Oh E.S., Alaniz L., Götte M. (2019). Proteoglycans and glycosaminoglycans as regulators of cancer stem cell function and therapeutic resistance. FEBS J..

[4] Vigetti D., Karousou E., Viola M., Passi A. (2015). Analysis of hyaluronan synthase activity. Methods Mol. Biol..

[5] Iozzo R.V., Schaefer L. (2015). Proteoglycan form and function: A comprehensive nomenclature of proteoglycans. Matrix Biol..

[6] Gotte M., Kersting C., Ruggiero M., Tio J., Tulusan A.H., Kiesel L., Wulfing P. (2006). Predictive value of syndecan-1 expression for the response to neoadjuvant chemotherapy of primary breast cancer. Anticancer. Res..

[7] Götte M., Yip G.W. (2006). Heparanase, hyaluronan, and CD44 in cancers: A breast carcinoma perspective. Cancer Res..

[8] Ibrahim S.A., Gadalla R., El-Ghonaimy E.A., Samir O., Mohamed H.T., Hassan H., Greve B., El-Shinawi M., Mohamed M.M., Gotte M. (2017). Syndecan-1 is a novel molecular marker for triple negative inflammatory breast cancer and modulates the cancer stem cell phenotype via the IL-6/STAT3, Notch and EGFR signaling pathways. Mol. Cancer.

[9] Kim C.W., Goldberger O.A., Gallo R.L., Bernfield M. (1994). Members of the syndecan family of heparan sulfate proteoglycans are expressed in distinct cell-, tissue-, and development-specific patterns. Mol. Biol. Cell.

[10] Teixeira F., Gotte M. (2020). Involvement of syndecan-1 and heparanase in cancer and inflammation. Adv. Exp. Med. Biol..

[11] Gotte M., Kersting C., Radke I., Kiesel L., Wulfing P. (2007). An expression signature of syndecan-1 (CD138), E-cadherin and c-met is associated with factors of angiogenesis and lymphangiogenesis in ductal breast carcinoma in situ. Breast Cancer Res..

[12] Leivonen M., Lundin J., Nordling S., von Boguslawski K., Haglund C. (2004). Prognostic value of syndecan-1 expression in breast cancer. Oncology.

[13] Baba F., Swartz K., van Buren R., Eickhoff J., Zhang Y., Wolberg W., Friedl A. (2006). Syndecan-1 and syndecan-4 are overexpressed in an estrogen receptor-negative, highly proliferative breast carcinoma subtype. Breast Cancer Res. Treat..

[14] Barbareschi M., Maisonneuve P., Aldovini D., Cangi M.G., Pecciarini L., Angelo Mauri F., Veronese S., Caffo O., Lucenti A., Palma P.D. (2003). High syndecan-1 expression in breast carcinoma is related to an aggressive phenotype and to poorer prognosis. Cancer.

[15] Hassan H., Greve B., Pavao M.S., Kiesel L., Ibrahim S.A., Gotte M. (2013). Syndecan-1 modulates beta-integrin-dependent and interleukin-6-dependent functions in breast cancer cell adhesion, migration, and resistance to irradiation. FEBS J..

[16] Ibrahim S.A., Hassan H., Vilardo L., Kumar S.K., Kumar A.V., Kelsch R., Schneider C., Kiesel L., Eich H.T., Zucchi I. (2013). Syndecan-1 (CD138) modulates triple-negative breast cancer stem cell properties via regulation of LRP-6 and IL-6-mediated STAT3 signaling. PLoS ONE.

[17] Ibrahim S.A., Yip G.W., Stock C., Pan J.W., Neubauer C., Poeter M., Pupjalis D., Koo C.Y., Kelsch R., Schule R. (2012). Targeting of syndecan-1 by microRNA miR-10b promotes breast cancer cell motility and invasiveness via a Rho-GTPase- and E-cadherin-dependent mechanism. Int. J. Cancer.

[18] Nikolova V., Koo C.Y., Ibrahim S.A., Wang Z., Spillmann D., Dreier R., Kelsch R., Fischgrabe J., Smollich M., Rossi L.H. (2009). Differential roles for membrane-bound and soluble syndecan-1 (CD138) in breast cancer progression. Carcinogenesis.

[19] Saleh M.E., Gadalla R., Hassan H., Afifi A., Gotte M., El-Shinawi M., Mohamed M.M., Ibrahim S.A. (2019). The immunomodulatory role of tumor Syndecan-1 (CD138) on ex vivo tumor microenvironmental CD4+ T cell polarization in inflammatory and non-inflammatory breast cancer patients. PLoS ONE.

[20] Viola M., Vigetti D., Karousou E., D’Angelo M.L., Caon I., Moretto P., De Luca G., Passi A. (2015). Biology and biotechnology of hyaluronan. Glycoconj J..

[21] Spinelli F.M., Vitale D.L., Sevic I., Alaniz L. (2020). Hyaluronan in the Tumor Microenvironment. Adv. Exp. Med. Biol..

[22] Boregowda R.K., Appaiah H.N., Siddaiah M., Kumarswamy S.B., Sunila S., Thimmaiah K.N., Mortha K., Toole B., Banerjee S. (2006). Expression of hyaluronan in human tumor progression. J. Carcinog.

[23] Auvinen P.K., Parkkinen J.J., Johansson R.T., Agren U.M., Tammi R.H., Eskelinen M.J., Kosma V.M. (1997). Expression of hyaluronan in benign and malignant breast lesions. Int. J. Cancer.

[24] Masarwah A., Tammi M., Sudah M., Sutela A., Oikari S., Kosma V.M., Tammi R., Vanninen R., Auvinen P. (2015). The reciprocal association between mammographic breast density, hyaluronan synthesis and patient outcome. Breast Cancer Res. Treat..

[25] Viola M., Bruggemann K., Karousou E., Caon I., Carava E., Vigetti D., Greve B., Stock C., De Luca G., Passi A. (2017). MDA-MB-231 breast cancer cell viability, motility and matrix adhesion are regulated by a complex interplay of heparan sulfate, chondroitin-/dermatan sulfate and hyaluronan biosynthesis. Glycoconj J..

[26] Hanahan D., Weinberg R.A. (2011). Hallmarks of cancer: The next generation. Cell.

[27] Pickup M.W., Mouw J.K., Weaver V.M. (2014). The extracellular matrix modulates the hallmarks of cancer. EMBO Rep..

[28] Sancho P., Barneda D., Heeschen C. (2016). Hallmarks of cancer stem cell metabolism. Br. J. Cancer.

[29] Greve B., Kelsch R., Spaniol K., Eich H.T., Gotte M. (2012). Flow cytometry in cancer stem cell analysis and separation. Cytom. A.

[30] Caon I., Bartolini B., Parnigoni A., Carava E., Moretto P., Viola M., Karousou E., Vigetti D., Passi A. (2020). Revisiting the hallmarks of cancer: The role of hyaluronan. Semin. Cancer Biol..

[31] Liu X., Guan Y., Wang L., Niu Y. (2017). MicroRNA-10b expression in node-negative breast cancer-correlation with metastasis and angiogenesis. Oncol. Lett..

[32] Piperigkou Z., Franchi M., Gotte M., Karamanos N.K. (2017). Estrogen receptor beta as epigenetic mediator of miR-10b and miR-145 in mammary cancer. Matrix Biol..

[33] Bourguignon L.Y., Wong G., Earle C., Krueger K., Spevak C.C. (2010). Hyaluronan-CD44 interaction promotes c-Src-mediated twist signaling, microRNA-10b expression, and RhoA/RhoC up-regulation, leading to Rho-kinase-associated cytoskeleton activation and breast tumor cell invasion. J. Biol. Chem..

[34] Szklarczyk D., Gable A.L., Lyon D., Junge A., Wyder S., Huerta-Cepas J., Simonovic M., Doncheva N.T., Morris J.H., Bork P. (2019). STRING v11: Protein-protein association networks with increased coverage, supporting functional discovery in genome-wide experimental datasets. Nucleic Acids Res..

[35] Bartha A., Gyorffy B. (2021). TNMplot.com: A web tool for the comparison of gene expression in normal, tumor and metastatic tissues. Int. J. Mol. Sci..

[36] Gyorffy B., Lanczky A., Eklund A.C., Denkert C., Budczies J., Li Q., Szallasi Z. (2010). An online survival analysis tool to rapidly assess the effect of 22,277 genes on breast cancer prognosis using microarray data of 1809 patients. Breast Cancer Res. Treat..

[37] Karalis T.T., Heldin P., Vynios D.H., Neill T., Buraschi S., Iozzo R.V., Karamanos N.K., Skandalis S.S. (2019). Tumor-suppressive functions of 4-MU on breast cancer cells of different ER status: Regulation of hyaluronan/HAS2/CD44 and specific matrix effectors. Matrix Biol..

[38] Wu M., Cao M., He Y., Liu Y., Yang C., Du Y., Wang W., Gao F. (2015). A novel role of low molecular weight hyaluronan in breast cancer metastasis. FASEB J..

[39] Sevic I., Spinelli F.M., Vitale D.L., Icardi A., Romano L., Brandone A., Giannoni P., Cristina C., Bolontrade M.F., Alaniz L. (2020). Hyaluronan metabolism is associated with DNA repair genes in breast and colorectal cancer. Screening of potential progression markers using qPCR. Biomedicines.

[40] Hamilton S.R., Fard S.F., Paiwand F.F., Tolg C., Veiseh M., Wang C., McCarthy J.B., Bissell M.J., Koropatnick J., Turley E.A. (2007). The hyaluronan receptors CD44 and Rhamm (CD168) form complexes with ERK1,2 that sustain high basal motility in breast cancer cells. J. Biol. Chem..

[41] Hassan N., Greve B., Espinoza-Sanchez N.A., Gotte M. (2021). Cell-surface heparan sulfate proteoglycans as multifunctional integrators of signaling in cancer. Cell Signal..

[42] Chalhoub N., Baker S.J. (2009). PTEN and the PI3-kinase pathway in cancer. Annu. Rev. Pathol..

[43] Karousou E., Misra S., Ghatak S., Dobra K., Gotte M., Vigetti D., Passi A., Karamanos N.K., Skandalis S.S. (2017). Roles and targeting of the HAS/hyaluronan/CD44 molecular system in cancer. Matrix Biol..

[44] Bourguignon L.Y., Singleton P.A., Zhu H., Diedrich F. (2003). Hyaluronan-mediated CD44 interaction with RhoGEF and Rho kinase promotes Grb2-associated binder-1 phosphorylation and phosphatidylinositol 3-kinase signaling leading to cytokine (macrophage-colony stimulating factor) production and breast tumor progression. J. Biol. Chem..

[45] Bernert B., Porsch H., Heldin P. (2011). Hyaluronan synthase 2 (HAS2) promotes breast cancer cell invasion by suppression of tissue metalloproteinase inhibitor 1 (TIMP-1). J. Biol. Chem..

[46] Garantziotis S., Savani R.C. (2019). Hyaluronan biology: A complex balancing act of structure, function, location and context. Matrix Biol..

[47] Bahena-Ocampo I., Espinosa M., Ceballos-Cancino G., Lizarraga F., Campos-Arroyo D., Schwarz A., Maldonado V., Melendez-Zajgla J., Garcia-Lopez P. (2016). miR-10b expression in breast cancer stem cells supports self-renewal through negative PTEN regulation and sustained AKT activation. EMBO Rep..

[48] Kang D., Jung S.H., Lee G.H., Lee S., Park H.J., Ko Y.G., Kim Y.N., Lee J.S. (2020). Sulfated syndecan 1 is critical to preventing cellular senescence by modulating fibroblast growth factor receptor endocytosis. FASEB J..

[49] Takahashi K., Yamanaka S. (2016). A decade of transcription factor-mediated reprogramming to pluripotency. Nat. Rev. Mol. Cell Biol..

[50] Troschel F.M., Minte A., Ismail Y.M., Kamal A., Abdullah M.S., Ahmed S.H., Deffner M., Kemper B., Kiesel L., Eich H.T. (2020). Knockdown of musashi RNA binding proteins decreases radioresistance but enhances cell motility and invasion in triple-negative breast cancer. Int. J. Mol. Sci..

[51] Spinelli F.M., Vitale D.L., Icardi A., Caon I., Brandone A., Giannoni P., Saturno V., Passi A., Garcia M., Sevic I. (2019). Hyaluronan preconditioning of monocytes/macrophages affects their angiogenic behavior and regulation of TSG-6 expression in a tumor type-specific manner. FEBS J..

[52] Vitale D.L., Spinelli F.M., Del Dago D., Icardi A., Demarchi G., Caon I., Garcia M., Bolontrade M.F., Passi A., Cristina C. (2018). Co-treatment of tumor cells with hyaluronan plus doxorubicin affects endothelial cell behavior independently of VEGF expression. Oncotarget.

[53] Maxwell C.A., Keats J.J., Crainie M., Sun X., Yen T., Shibuya E., Hendzel M., Chan G., Pilarski L.M. (2003). RHAMM is a centrosomal protein that interacts with dynein and maintains spindle pole stability. Mol. Biol. Cell.

[54] Sun H., Berquin I.M., Owens R.T., O’Flaherty J.T., Edwards I.J. (2008). Peroxisome proliferator-activated receptor gamma-mediated up-regulation of syndecan-1 by n-3 fatty acids promotes apoptosis of human breast cancer cells. Cancer Res..

[55] Sun H., Hu Y., Gu Z., Owens R.T., Chen Y.Q., Edwards I.J. (2011). Omega-3 fatty acids induce apoptosis in human breast cancer cells and mouse mammary tissue through syndecan-1 inhibition of the MEK-Erk pathway. Carcinogenesis.

[56] Bui N.L., Pandey V., Zhu T., Ma L., Basappa, Lobie P.E. (2018). Bad phosphorylation as a target of inhibition in oncology. Cancer Lett..

[57] Thompson E.B. (1998). The many roles of c-Myc in apoptosis. Annu. Rev. Physiol..

[58] Elenius V., Gotte M., Reizes O., Elenius K., Bernfield M. (2004). Inhibition by the soluble syndecan-1 ectodomains delays wound repair in mice overexpressing syndecan-1. J. Biol. Chem..

[59] Gotte M., Joussen A.M., Klein C., Andre P., Wagner D.D., Hinkes M.T., Kirchhof B., Adamis A.P., Bernfield M. (2002). Role of syndecan-1 in leukocyte-endothelial interactions in the ocular vasculature. Investig. Ophthalmol. Vis. Sci..

[60] Rapraeger A.C. (2013). Synstatin: A selective inhibitor of the syndecan-1-coupled IGF1R-alphavbeta3 integrin complex in tumorigenesis and angiogenesis. FEBS J..

[61] Theocharis A.D., Skandalis S.S., Neill T., Multhaupt H.A., Hubo M., Frey H., Gopal S., Gomes A., Afratis N., Lim H.C. (2015). Insights into the key roles of proteoglycans in breast cancer biology and translational medicine. Biochim. Biophys. Acta.

[62] Iozzo R.V., Sanderson R.D. (2011). Proteoglycans in cancer biology, tumour microenvironment and angiogenesis. J. Cell Mol. Med..

