# Supplementary material for: Syndecan-1 Depletion Has a Differential Impact on Hyaluronic Acid Metabolism and Tumor Cell Behavior in Luminal and Triple-Negative Breast Cancer Cells
Source: Int J Mol Sci. 2021 May 30;22(11):5874. doi: 10.3390/ijms22115874 (PMC8198019; doi:10.3390/ijms22115874)
Supplement: Supplementary file 1 [file ijms-22-05874-s001.zip › ijms-1220310-supplementary.pdf]

Supplementary Files. Syndecan-1 depletion has a differential impact on hyaluronic acid metabolism and tumor cell behavior in luminal and triple negative breast cancer cells.

Valla SA, Hassan N, Vitale DL, Madanes D, Spinelli FM, Texeira FCOB, Greve B, Espinoza-Sánchez NA, Cristina C, Alaniz L, Götte M

Supplementary Figures

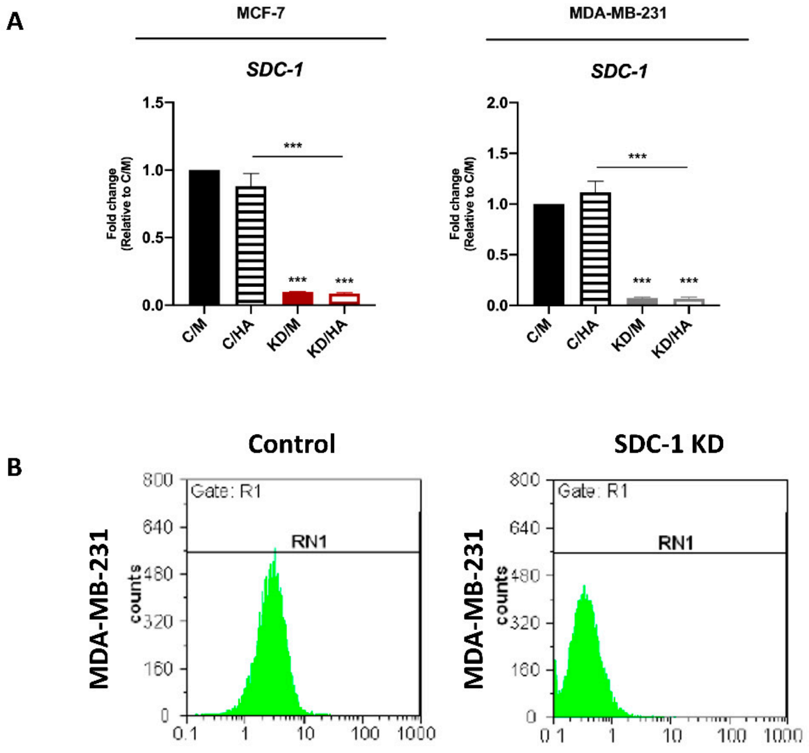

<u>Supplementary Figure 1.</u> **siRNA transfection against Sdc-1 reduced its mRNA expression in 95% in both MDA-MB-231 and MCF-7 cells. A)** *Sdc-1* expression was determined by RT-qPCR after 48 hours post-transfection, normalized to *β-ACTIN* expression and plotted as fold of C/M. B) Protein levels of Sdc-1 as determined by flow cytometry in control and Sdc-1 KD MDA-MB-231 cells. Representative images are shown. Data represent the mean±SEM from 3 independent experiments. Asterisks represent comparisons with statistically significant differences (**p<0,01).

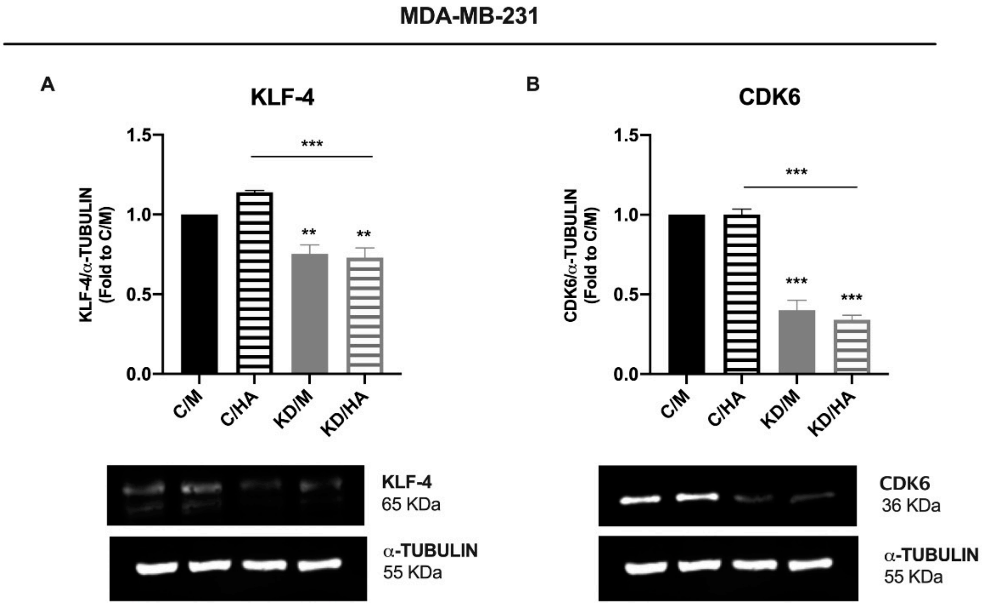

Supplementary Figure 2. Sdc-1-deficient MDA-MB-231 cells have reduced levels of KLF-4 and CDK6 proteins. KLF-4 (A) and CDK6 (B) protein levels were detected by Western Blot, normalized to α-TUBULIN levels, and plotted as fold of C/M. Representative bands are shown. Data represent the mean±SEM from 4 independent experiments. Asterisks represent comparisons with statistically significant differences (*p<0,05, **p<0,01).

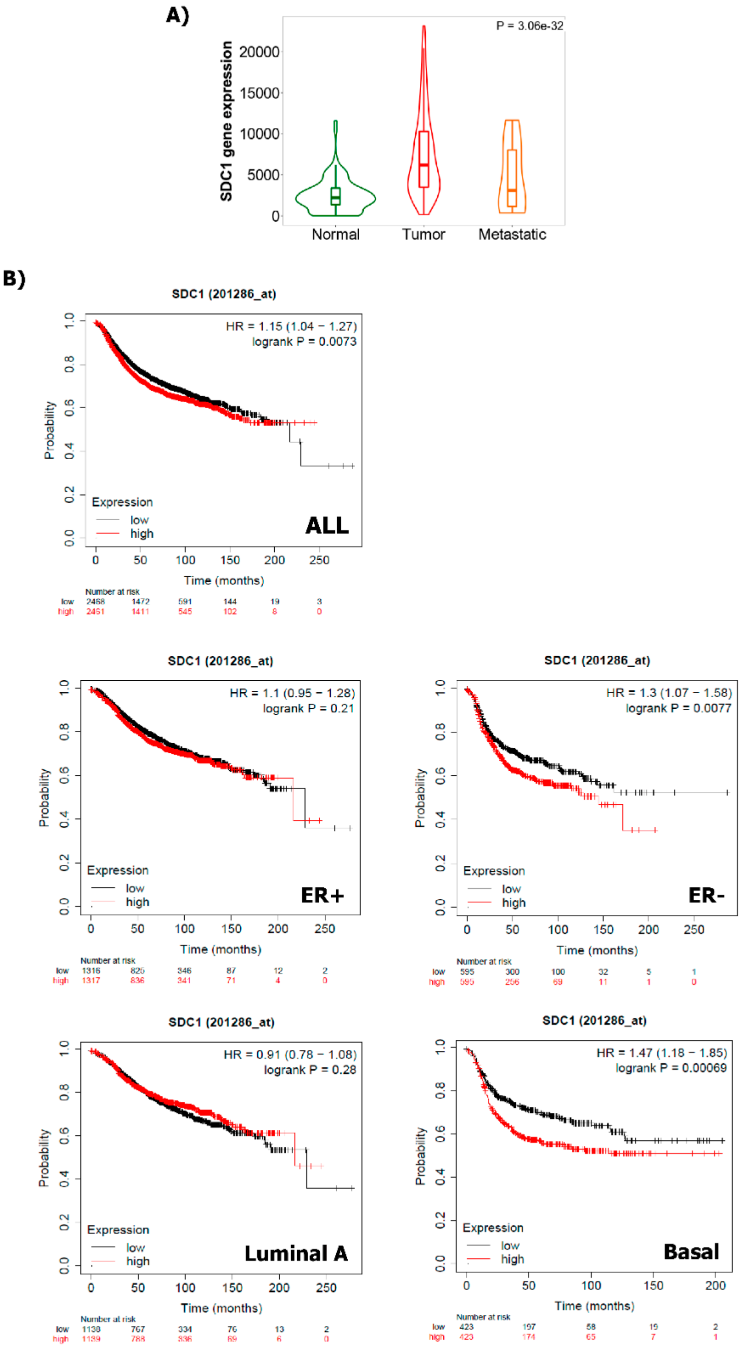

<u>Supplementary Figure 3.</u> The differential expression and the prognostic value of Sdc-1 in tumor tissues of breast cancer patients. (A) Violin plots of Sdc-1 gene expression in non-tumor, primary and metastatic breast cancer tumor samples. The statistical significance cutoff was set at $p < 0.01$. (B) Kaplan-Meier relapse-free survival curves are plotted for breast cancer patients with unfiltered patients (All), and patients of the estrogen receptor-positive (ER+), estrogen receptor-negative (ER-), Luminal A, and Basal subtypes and the expression of Sdc-1. Log-rank p values and hazard ratios (HRs; 95 % confidence interval in parentheses) are shown.

Supplementary Tables.

Supplementary Table 1. GO enrichment analysis associated with SDC-1 and proteins of interest investigated in this study. The STRING program was used to perform this analysis.

Category	Pathway description	Observed gene count	False discovery rate (FDR)	Matching proteins in your network (labels)
Biological Process	GO:0006027-glycosaminoglycan catabolic process	15	2.06E-21	DCN,GPC1,FGF2,GPC2,SDC2,BGN,SDC3,GPC4,SDC4,HSPG2,GPC6,GPC5,AGRN,SDC1,GPC3
	GO:0030203-glycosaminoglycan metabolic process	17	5.91E-20	DCN,HGF,GPC1,FGF2,GPC2,HAS2,SDC2,BGN,SDC3,GPC4,SDC4,HSPG2,GPC6,GPC5,AGRN,SDC1,GPC3
	GO:0006024-glycosaminoglycan biosynthetic process	15	3.00E-19	DCN,GPC1,GPC2,HAS2,SDC2,BGN,SDC3,GPC4,SDC4,HSPG2,GPC6,GPC5,AGRN,SDC1,GPC3
	GO:0009653-anatomical structure morphogenesis	33	9.26E-18	DCN,EFNB1,MAPK1,HGF,SERPINE1,NANOG,EFNB2,POU5F1,THBS1,GPC1,FGF2,STAT3,EGF,S100B,TDGF1,SHH,HAS2,SDC2,MET,SOX2,FGFR3,FN1,SDC4,SRC,HSPG2,SDC1,GPC3,ITGB1,FGFR1,FGFR2,DAG1,VEGFA,FGF1
	GO:0007166-cell surface receptor signaling pathway	34	1.17E-17	DCN,EFNB1,MAPK1,HGF,NANOG,EFNB2,POU5F1,SDCBP,FGF2,STAT3,EGF,GPC2,FGFR4,SHH,IGF1,SDC2,MET,SOX2,BGN,FGFR3,FN1,GPC4,SRC,GPC6,SDC1,FGFBP1,AFP,ITGB1,NOTUM,FGFR1,FGFR2,IL5RA,VEGFA,FGF1
	GO:0050793-regulation of developmental process	34	2.02E-16	DCN,MAPK1,HGF,SERPINE1,NANOG,EFNB2,POU5F1,SDCBP,THBS1,GPC1,FGF2,STAT3,EGF,S100B,SHH,IGF1,HAS2,SDC2,SOX2,FGFR3,FN1,GPC4,SRC,HSPG2,GPC6,AGRN,FGFBP1,GPC3,ITGB1,FGFR1,FGFR2,DAG1,VEGFA,FGF1
	GO:0051239-regulation of multicellular organismal process	35	1.22E-15	DCN,MAPK1,HGF,SERPINE1,EFNB2,POU5F1,SDCBP,THBS1,GPC1,FGF2,STAT3,EGF,S100B,TDGF1,SHH,IGF1,HAS2,SDC2,MET,SOX2,FN1,GPC4,SRC,HSPG2,

				GPC6,AGRN,FGFBP1,GPC3,ITGB1,FGFR1,FGFR2,IL5RA,DAG1,VEGFA,FGF1
	GO:0022603-regulation of anatomical structure morphogenesis	24	1.26E-15	DCN,HGF,SERPINE1,POU5F1,THBS1,FGF2,STAT3,S100B,SHH,HAS2,SDC2,FN1,GPC4,SRC,HSPG2,GPC6,FGFBP1,GPC3,ITGB1,FGFR1,FGFR2,DAG1,VEGFA,FGF1
	GO:0030334-regulation of cell migration	22	1.84E-15	DCN,MAPK1,HGF,SERPINE1,SDCBP,THBS1,FGF2,STAT3,EGF,TDGF1,SHH,IGF1,HAS2,MET,FN1,SDC4,SRC,FGFBP1,FGFR1,DAG1,VEGFA,FGF1
	GO:2000026-regulation of multicellular organismal development	30	2.11E-15	DCN,HGF,SERPINE1,EFNB2,POU5F1,SDCBP,THBS1,GPC1,FGF2,STAT3,EGF,S100B,SHH,IGF1,SDC2,SOX2,FN1,GPC4,SRC,HSPG2,GPC6,AGRN,FGFBP1,GPC3,ITGB1,FGFR1,FGFR2,DAG1,VEGFA,FGF1
**Molecular Function**	GO:0050840-extracellular matrix binding	10	1.48E-13	DCN,THBS1,GPC1,NID1,SHH,BGN,AGRN,ITGB1,DAG1,VEGFA
	GO:0004713-protein tyrosine kinase activity	13	3.25E-13	EFNB1,HGF,EFNB2,FGF2,EGF,FGFR4,MET,FGFR3,SRC,FGFR1,FGFR2,IL5RA,FGF1
	GO:0046934-phosphatidylinositol-4,5-bisphosphate 3-kinase activity	10	6.66E-13	HGF,FGF2,EGF,FGFR4,MET,FGFR3,SRC,FGFR1,FGFR2,FGF1
	GO:0005539-glycosaminoglycan binding	13	1.25E-12	DCN,THBS1,FGF2,FGFR4,SHH,BGN,FN1,AGRN,FGFBP1,FGFR1,FGFR2,VEGFA,FGF1
	GO:0017134-fibroblast growth factor binding	7	2.60E-11	THBS1,GPC1,FGFR4,FGFR3,FGFBP1,FGFR1,FGFR2
	GO:0043236-laminin binding	7	1.28E-10	THBS1,GPC1,NID1,SHH,AGRN,ITGB1,DAG1
	GO:0008201-heparin binding	9	1.76E-08	THBS1,FGF2,FGFR4,FN1,FGFBP1,FGFR1,FGFR2,VEGFA,FGF1
	GO:0005102-signaling receptor binding	20	1.88E-08	EFNB1,HGF,SERPINE1,EFNB2,SDCBP,THBS1,FGF2,STAT3,EGF,S100B,TDGF1,SHH,IGF1,FN1,SRC,HSPG2,ITGB1,FGFR1,VEGFA,FGF1
	GO:1901681-sulfur compound binding	10	1.88E-08	THBS1,FGF2,FGFR4,FN1,AGRN,FGFBP1,FGFR1,FGFR2,VEGFA,FGF1

	GO:0004672-protein kinase activity	14	2.01E-08	EFNB1,MAPK1,HGF,EFNB2,FGF2,EGF,FGFR4,MET,FGFR3,SRC,FGFR1,FGFR2,IL5RA,FGF1
**Cellular Component**	GO:0005576-extracellular region	38	9.08E-21	MAPK1,NID2,HGF,SERPINE1,SDCBP,THBS1,CDH1,GPC1,NID1,FGF2,EGF,S100B,GPC2,FGFR4,ALB,TDGF1,SHH,IGF1,MET,BGN,FGFR3,FN1,GPC4,SDC4,HSPG2,GPC6,GPC5,AGRN,FGFBP1,GPC3,AFP,NOTUM,FGFR1,FGFR2,IL5RA,DAG1,VEGFA,FGF1
	GO:0005796-Golgi lumen	15	6.08E-20	DCN,GPC1,GPC2,SDC2,BGN,SDC3,GPC4,SDC4,HSPG2,GPC6,GPC5,AGRN,SDC1,GPC3,DAG1
	GO:0043202-lysosomal lumen	14	1.07E-18	DCN,GPC1,GPC2,SDC2,BGN,SDC3,GPC4,SDC4,HSPG2,GPC6,GPC5,AGRN,SDC1,GPC3
	GO:0005775-vacuolar lumen	16	1.12E-18	DCN,MAPK1,SDCBP,GPC1,GPC2,SDC2,BGN,SDC3,GPC4,SDC4,HSPG2,GPC6,GPC5,AGRN,SDC1,GPC3
	GO:0031012-extracellular matrix	18	1.57E-18	NID2,SERPINE1,THBS1,GPC1,NID1,GPC2,SHH,BGN,FN1,GPC4,HSPG2,GPC6,GPC5,AGRN,GPC3,DAG1,VEGFA,FGF1
	GO:0005615-extracellular space	23	1.07E-13	HGF,SERPINE1,SDCBP,THBS1,GPC1,FGF2,EGF,S100B,GPC2,ALB,TDGF1,SHH,IGF1,FN1,GPC6,GPC5,FGFBP1,GPC3,AFP,IL5RA,DAG1,VEGFA,FGF1
	GO:0062023-collagen-containing extracellular matrix	12	2.05E-13	NID2,SERPINE1,NID1,GPC2,FN1,GPC4,HSPG2,GPC6,GPC5,AGRN,GPC3,DAG1
	GO:0071944-cell periphery	41	2.52E-13	EFNB1,MAPK1,NID2,SERPINE1,EFNB2,SDCBP,THBS1,CDH1,GPC1,NID1,STAT3,EGF,GPC2,FGFR4,TDGF1,SHH,IGF1,HAS2,SDC2,MET,BGN,FGFR3,SDC3,FN1,GPC4,SDC4,SRC,HSPG2,GPC6,GPC5,AGRN,SDC1,FGFBP1,GOLM1,GPC3,ITGB1,FGFR1,FGFR2,IL5RA,DAG1,FGF1
	GO:0005764-lysosome	17	2.77E-12	DCN,MAPK1,SDCBP,GPC1,GPC2,SDC2,BGN,SDC3,GPC4,SDC4,SRC,HSPG2,GPC6,GPC5,AGRN,SDC1,GPC3
	GO:0005886-plasma membrane	39	7.73E-12	EFNB1,MAPK1,NID2,SERPINE1,EFNB2,SDCBP,THBS1,CDH1,GPC1,STAT3,EGF,GPC2,FGFR4,TDGF1,SHH,

				IGF1,HAS2,SDC2,MET,BGN,FGFR3,SDC3,FN1,GPC4,SDC4,SRC,HSPG2,GPC6,GPC5,AGRN,SDC1,FGFBP1,GOLM1,GPC3,ITGB1,FGFR1,FGFR2,IL5RA,DAG1
**KEEG enrichment analysis**	hsa05205-Proteoglycans in cancer	20	3.37E-24	DCN,MAPK1,HGF,NANOG,THBS1,GPC1,FGF2,STAT3,SHH,IGF1,SDC2,MET,SDC4,SRC,HSPG2,SDC1,GPC3,ITGB1,FGFR1,VEGFA
	hsa04015-Rap1 signaling pathway	16	1.74E-17	MAPK1,HGF,THBS1,CDH1,FGF2,EGF,FGFR4,IGF1,MET,FGFR3,SRC,ITGB1,FGFR1,FGFR2,VEGFA,FGF1
	hsa01521-EGFR tyrosine kinase inhibitor resistance	11	2.19E-14	MAPK1,HGF,FGF2,STAT3,EGF,IGF1,MET,FGFR3,SRC,FGFR2,VEGFA
	hsa05200-Pathways in cancer	17	5.88E-13	MAPK1,HGF,CDH1,FGF2,STAT3,EGF,FGFR4,SHH,IGF1,MET,FGFR3,ITGB1,FGFR1,FGFR2,IL5RA,VEGFA,FGF1
	hsa04550-Signaling pathways regulating pluripotency of stem cells	11	4.42E-12	MAPK1,NANOG,POU5F1,FGF2,STAT3,FGFR4,IGF1,SOX2,FGFR3,FGFR1,FGFR2
	hsa04151-PI3K-Akt signaling pathway	14	9.24E-12	MAPK1,HGF,THBS1,FGF2,EGF,FGFR4,IGF1,MET,FGFR3,ITGB1,FGFR1,FGFR2,VEGFA,FGF1
	hsa05218-Melanoma	9	1.44E-11	MAPK1,HGF,CDH1,FGF2,EGF,IGF1,MET,FGFR1,FGF1
	hsa04014-Ras signaling pathway	12	2.04E-11	MAPK1,HGF,FGF2,EGF,FGFR4,IGF1,MET,FGFR3,FGFR1,FGFR2,VEGFA,FGF1
	hsa04010-MAPK signaling pathway	12	3.02E-10	MAPK1,HGF,FGF2,EGF,FGFR4,IGF1,MET,FGFR3,FGFR1,FGFR2,VEGFA,FGF1
	hsa05219-Bladder cancer	7	5.85E-10	MAPK1,THBS1,CDH1,EGF,FGFR3,SRC,VEGFA

Supplementary Table 2. Statistical co-citation analysis associated with SDC-1 and proteins of interest investigated in this study. The STRING program was used to perform this analysis.

Pathway Description	Observed gene count	False discovery rate	Matching proteins in your network (labels)
(2015) Insights into the key roles of proteoglycans in breast cancer biology and translational medicine.	26	2.76E-35	DCN,HGF,SDCBP,THBS1,CDH1,GPC1,FGF2,STAT3,EGF,IGF1,SDC2,BGN,SDC3,FN1,SDC4,SRC,HSPG2,GPC6,GPC5,AGRN,SDC1,GPC3,ITGB1,NOTUM,FGFR1,VEGFA
(2015) Fell-Muir Lecture: Heparan sulphate and the art of cell regulation: a polymer chain conducts the protein orchestra.	22	5.29E-33	GPC1,FGF2,GPC2,SHH,SDC2,MET,FGFR3,SDC3,FN1,GPC4,SDC4,SRC,HSPG2,GPC6,GPC5,AGRN,SDC1,GPC3,NOTUM,FGFR1,VEGFA,FGF1
(2010) Novel aspects of corneal angiogenic and lymphangiogenic privilege.	23	2.49E-32	DCN,EFNB1,EFNB2,THBS1,CDH1,GPC1,NID1,FGF2,STAT3,GPC2,ALB,BGN,FN1,GPC4,SRC,HSPG2,GPC6,GPC5,SDC1,GPC3,FGFR1,VEGFA,FGF1
(2017) Exploiting Heparan Sulfate Proteoglycans in Human Neurogenesis-Controlling Lineage Specification and Fate.	20	5.31E-31	GPC1,FGF2,EGF,S100B,GPC2,SHH,SDC2,SOX2,SDC3,GPC4,SDC4,SRC,HSPG2,GPC6,GPC5,AGRN,SDC1,GPC3,VEGFA,FGF1
(2014) Syndecans as modulators and potential pharmacological targets in cancer progression.	20	5.31E-31	HGF,THBS1,CDH1,GPC1,FGF2,EGF,GPC2,SDC2,SDC3,FN1,GPC4,SDC4,HSPG2,GPC6,GPC5,AGRN,SDC1,GPC3,FGFR1,VEGFA
(2019) Spotlight on the Transglutaminase 2-Heparan Sulfate Interaction.	18	1.14E-30	SDCBP,GPC1,FGF2,GPC2,SHH,SDC2,SDC3,FN1,GPC4,SDC4,SRC,HSPG2,GPC6,GPC5,AGRN,GPC3,ITGB1,VEGFA
(2008) Heparan sulfate proteoglycans: a GAGgle of skeletal-hematopoietic regulators.	20	1.14E-30	DCN,GPC1,NID1,FGF2,GPC2,SHH,SDC2,BGN,FGFR3,SDC3,FN1,GPC4,HSPG2,GPC6,GPC5,SDC1,GPC3,DAG1,VEGFA,FGF1
(2007) The extracellular matrix and blood vessel formation: not just a scaffold.	20	1.54E-30	NID2,THBS1,GPC1,NID1,FGF2,EGF,GPC2,SDC2,SDC3,FN1,GPC4,SDC4,SRC,HSPG2,GPC6,GPC5,AGRN,SDC1,GPC3,VEGFA
(2011) Proteoglycans in cancer biology, tumour microenvironment and angiogenesis.	20	1.80E-30	DCN,HGF,CDH1,GPC1,FGF2,EGF,GPC2,SDC2,FN1,GPC4,SDC4,SRC,HSPG2,GPC6,GPC5,SDC1,GPC3,FGFR1,VEGFA,FGF1

(2014) Matrix regulators in neural stem cell functions.	18	1.34E-29	CDH1,GPC1,FGF2,EGF,GPC2,SHH,FGFR3,SDC3,FN1,GPC4,SDC4,HSPG2,GPC6,GPC5,SDC1,GPC3,FGFR1,VEGFA

Supplementary Table 3. List of primers and probes used for RT-qPCR.

Primer ID		Sequence
**SYBR Green primers**		
**HAS-2**	***Forward***	TACACAGCCTTCAGAGCACTG
	Reverse	ATGAGGCTGGGTCAAGCATAG
**RHAMM**	***Forward***	TGGAAAAGATGGAAGCAAGG
	Reverse	CCAGTGTAGCATTATTTGCAGAG
**PDGF-A**	***Forward***	TGCCTCTCCGCACTCACTG
	Reverse	AGAACATGGGCGAGGTATCC
**ACTB**	***Forward***	TCAAGATCATTGCTCCTCCTGAG
	Reverse	ACATCTGCTGGAAGGTGGACA
**GAPDH**	***Forward***	GGGGCTGCCCAGAACATCAT
	Reverse	GCCTGCTTCACCACCTTCTTG
**SDC-1**	***Forward***	AGGACGAAGGCAGCTACTCCT
	Reverse	TTTGGTGGGCTTCTGGTAGG
**IL-8**	***Forward***	CAAGAGCCAGGAAGAAACCA
	Reverse	AGCACTCCTTGGCAAAACTG
**CD44**	***Forward***	GCCCATTGTTCATTCTTGTGCT
	Reverse	AAACCAGAGGAAGGGTGTGCTC
**CCND1**	***Forward***	GTCCTGTGACGCGCAAGTCT
	Reverse	ACATGTTGGTGCTGGGAAGC
**CD133**	***Forward***	TCAAAGATTGGCCATGTTCCAC
	Reverse	TGTCAGATGGAGTTACGCAGGTT
**OCT-4**	***Forward***	CTGGAGTTTGTGCCAGGGTTT
	Reverse	CTTCACCTTCCCTCCAACCAG
**TGF-β**	***Forward***	TACCTGAACCCGTGTTGCTCTC
	Reverse	GTTGCTGAGGTATCGCCAGGAA
**MYC-2**	***Forward***	CAACCTCACAACCTTGGCTGA
	Reverse	CCAAAGTCCAATTTGAGGCAGTT
**WNT3A**	***Forward***	AGGTCCCACAGCCCTGAGAT
	Reverse	TCCAGGAAAGCGGACCATTT
**WNT5A**	***Forward***	TCGTTAGCAGCATCAGTCCACA
	Reverse	GACCTGTGCCTTCGTGCCTA
**SFRP-1**	***Forward***	GATGCAGGAGGCTCAGGTGAT
	Reverse	GCTGGCAACAGGTCAGAACG
**KLF-4**	***Forward***	GCCTAAATGATGGTGCTTGGTG
	Reverse	GCAGTTTGAAAACTTTGGCTTCC
**TCF-7L1**	***Forward***	AAGGTGCCTGCCACTTCCTC
	Reverse	CCTGCCACTCTGGGATTGTG
**CCNB1**	***Forward***	TTTGGACAGTTTACCAGTTGCCTTT
	Reverse	CTCTTGAAGCARCGTATCACAGCA
**TWIST**	***Forward***	GCGGCCAGGTACATCGACTT
	Reverse	TGCAGCTTGCCATCTTGGAG
**SNAIL**	***Forward***	CGAGCCCAGGCAGCTATTTC
	Reverse	CCCGACAAGTGACAGCCATT
**MSI-2**	***Forward***	CCTAGTATGCTTGCCTCACAAACG
	Reverse	CGTCATCAGGGAGAAGCACAG
**Taqman probes**		
**ACTB**	***PROBE ID***	Hs 99999903 m1
**VEGFA**	***PROBE ID***	Hs 00900054 m1
**ANGP1**	***PROBE ID***	Hs 00181613 m1

**RNU44**	***PROBE ID***	001094
**hsa-miR-10b**	***PROBE ID***	002218

<u>Supplementary Table 4.</u> List of primary antibodies employed in Western Blots.

Antibody	Cat. no.	Supplier
**BAD**	#9292	Cell Signaling Technology
**BCL-2**	#2872	Cell Signaling Technology
**CDK6**	#13331	Cell Signaling Technology
**p-AKT**	#9271	Cell Signaling Technology
**p-ERK**	#9101	Cell Signaling Technology
**p-PTEN**	#9551	Cell Signaling Technology
**Tubulin**	sc-5286	Santa Cruz Biotechnology
